# A time-course comparative clinical and immune response evaluation study between the human pathogenic *Orientia tsutsugamushi* strains: Karp and Gilliam in a rhesus macaque (*Macaca mulatta*) model

**DOI:** 10.1371/journal.pntd.0010611

**Published:** 2022-08-04

**Authors:** Manutsanun Inthawong, Piyanate Sunyakumthorn, Sirima Wongwairot, Tippawan Anantatat, Susanna J. Dunachie, Rawiwan Im-Erbsin, James W. Jones, Carl J. Mason, Luis A. Lugo, Stuart D. Blacksell, Nicholas P. J. Day, Piengchan Sonthayanon, Allen L. Richards, Daniel H. Paris

**Affiliations:** 1 Department of Veterinary Medicine, United States Army Medical Directorate, Armed Forces Research Institute of Medical Sciences (USAMD-AFRIMS), Bangkok, Thailand; 2 Department of Molecular Tropical Medicine and Genetics, Faculty of Tropical Medicine, Mahidol University, Bangkok, Thailand; 3 Mahidol-Oxford Tropical Medicine Research Unit (MORU), Faculty of Tropical Medicine, Mahidol University, Bangkok, Thailand; 4 Department of Diagnostic Medicine/Pathobiology, College of Veterinary Medicine, Kansas State University, Manhattan, Kansas, United States of America; 5 Centre for Tropical Medicine and Global Health, Nuffield Department of Clinical Medicine, University of Oxford, United Kingdom; 6 Viral & Rickettsial Diseases Department, Naval Medical Research Center, Silver Spring, Maryland, United States of America; 7 Department of Preventive Medicine and Biostatistics, Uniformed Services University of the Health Sciences, Bethesda, Maryland, United States of America; 8 Department of Medicine, Swiss Tropical and Public Health Institute, Faculty of Medicine, University of Basel, Switzerland; 9 Department of Clinical Research, Faculty of Medicine, University of Basel, Switzerland; University of Texas Medical Branch, UNITED STATES

## Abstract

**Background:**

Scrub typhus is a vector-borne febrile illness caused by *Orientia tsutsugamushi* transmitted by the bite of Trombiculid mites. *O*. *tsutsugamushi* has a high genetic diversity and is increasingly recognized to have a wider global distribution than previously assumed.

**Methodology/principle findings:**

We evaluated the clinical outcomes and host immune responses of the two most relevant human pathogenic strains of *O*. *tsutsugamushi;* Karp (n = 4) and Gilliam (n = 4) in a time-course study over 80 days post infection (dpi) in a standardized scrub typhus non-human primate rhesus macaque model. We observed distinct features in clinical progression and immune response between the two strains; Gilliam-infected macaques developed more pronounced systemic infection characterized by an earlier onset of bacteremia, lymph node enlargement, eschar lesions and higher inflammatory markers during the acute phase of infection, when compared to the Karp strain. C-reactive protein (CRP) plasma levels, interferon gamma (IFN-γ, interleukin-1 receptor antagonist (IL-1ra), IL-15 serum concentrations, CRP/IL10- and IFN-γ/IL-10 ratios correlated positively with bacterial load in blood, implying activation of the innate immune response and preferential development of a T helper-type 1 immune response. The *O*. *tsutsugamushi*-specific immune memory responses in cells isolated from skin and lymph nodes at 80 dpi were more markedly elevated in the Gilliam-infected macaques than in the Karp-infected group. The comparative cytokine response dynamics of both strains revealed significant up-regulation of IFN-γ, tumor necrosis factor (TNF), IL-15, IL-6, IL-18, regulatory IL-1ra, IL-10, IL-8 and granulocyte-colony-stimulating factor (G-CSF). These data suggest that the clinical outcomes and host immune responses to scrub typhus could be associated with counter balancing effects of pro- and anti-inflammatory cytokine-mediated responses.

Currently, no data on characterized time-course comparisons of *O*. *tsutsugamushi* strains regarding measures of disease severity and immune response is available. Our study provides evidence for the strain-specificity of host responses in scrub typhus, which supports our understanding of processes at the initial inoculation site (eschar), systemic disease progression, protective and/or pathogenic host immune mechanisms and cellular immune memory function.

**Conclusions/significance:**

This study characterised an improved intradermal rhesus macaque challenge model for scrub typhus, whereby the Gilliam strain infection associated with higher disease severity in the rhesus macaque model than the previous Karp strain infection. Difficulties associated with inoculum quantitation for obligate-intracellular bacteria were overcome by using functional inoculum titrations in outbred mice. The Gilliam-based rhesus macaque model provides improved endpoint measurements and contributes towards the identification of correlates of protection for future vaccine development.

## Author summary

Scrub typhus is a mite-borne rickettsial illness, which is potentially severe if untreated or diagnosis is delayed or missed. This easily treatable disease is endemic in southeastern and eastern parts of Asia, but is increasingly recognised to have a global distribution. In endemic areas, where infected vectors transmit different strains of *Orientia tsutsugamushi—*the causative pathogen of scrub typhus—the diversity of these bacterial strains poses a serious obstacle for developing effective diagnostics and a universal scrub typhus vaccine. Comparison studies on the host immune responses against various strains are rare and there is limited evidence on strain-specific immune responses over the disease course.

In this study, we characterized the clinical disease progression from the start of infection to full convalescence in a well-characterised non-human primate rhesus macaque model, infected with the two most prevalent *O*. *tsutsugamushi* strains; Karp and Gillam, and compared the time course dynamics of bacteremia with correlations of various immune response mechanisms. We found that Gilliam strain infection associated with higher disease severity, earlier onset of bacteremia, lymph node enlargement, eschar lesions and higher inflammatory markers during the acute phase of infection, when compared to the Karp strain. The findings suggest that a Gilliam strain infection in rhesus macaques provides improved endpoint measurements when compared to Karp strain infections, which is useful for future vaccine development.

## Introduction

Scrub typhus remains one of the most neglected potentially severe, but easily-treatable diseases in (sub)-tropical countries [1,2]. The increasing number of reported cases of scrub typhus outside SE-Asia, emphasizes the need to raise awareness of the disease globally [3–5]. Scrub typhus causes an acute undifferentiated fever that is fatal in approx. 6–8% of untreated cases and in 1.4% in treated cases (median mortality) [2,6]. The etiological agent *Orientia tsutsugamushi*, an obligate intracellular Gram-negative bacteria, transmitted to humans through the bite of the infected larval stage of Trombiculid mites called chiggers. The broad genetic diversity of *O*. *tsutsugamushi* isolates recognized in endemic areas is exemplified by the hypervariable regions within the 56-kDa type specific antigen (TSA) gene [7,8]. Important knowledge gaps remain on associations between the immunogenicity and genetic variability of *O*. *tsutsugamushi* strains, disease severity and laboratory correlates of pathogenicity [7,9,10]. It is the aim of this study to address these in a validated non-human primate model.

In mouse models of scrub typhus, studies showed that the extent of *O*. *tsutsugamushi* pathogenicity was dependent on both the bacterial strains’ individual virulence factors and the mouse strains infected [11,12]. *O*. *tsutsugamushi* strains are classified into three virulence groups for mice: Low (KN-1, KN-2 and GJ-1), Intermediate (Gilliam) and highly virulent (Karp, Kato and KN-3) strains [12]. Intradermal inoculation of CD-1 Swiss outbred mice (closely mimicking the natural infection route) revealed that Karp and Gilliam strains led to more severe disease than Kato and Woods strains [13] and that the intracellular cytokine signatures induced by the intradermal inoculation of Karp and Woods strains (non-lethal scrub typhus models) differed significantly from that induced by the intraperitoneal inoculation of Karp strain (lethal scrub typhus model) [10]. Available data from mouse and human studies suggest that in severe scrub typhus with an overwhelming pathogen load, the host immune system produces a strong inflammatory cytokine response, which associates with increased disease severity and complications [10,14–17].

In non-human primates (NHPs), early studies suggest that the Gilliam strain associates with more severe disease and persistent antibody responses [18–20]. NHPs exhibit a closer response to scrub typhus infection to humans than mice, as NHPs develop a febrile illness and eschars at the inoculation site, while mice do not [13,21,22]. Silvered-leaf macaques inoculated intradermally with *O*. *tsutsugamushi* Karp, Gilliam and/or Kato strains (propagated in yolk sacs of embryonated chicken eggs) developed clinical illness and eschars identical to those seen in human scrub typhus, whereas inoculation with TA678 and TA686 strains of *O*. *tsutsugamushi*, showed little or no signs of disease [18,23]. The Gilliam strain was described as the most ‘virulent’ strain, as it associated with the highest antibody titers and most severe disease manifestations in Silvered-leaf and cynomolgus macaques. The availability, ease of maintenance, high similarity of NHP responses to humans, and accessibility to clinical-pathological responses (i.e. fever, eschar) supported NHP investigations—but to date, no lethal scrub typhus disease model has been developed specifically for NHPs.

In humans, the predominant representative strains responsible for symptomatic disease are Karp (65%) and Gilliam (26%). The original antigenic prototype strains of Karp, Gilliam and Kato remain the most commonly used strains for diagnostics and experimental studies (21–23). The concept of regional strain diversity remains controversial, and is likely influenced more by the mite in its role as vector and/or host, than by vertebrate hosts (mostly rodents)—but not by humans, as they represent dead-end hosts [24]. A recent study in Vietnam suggested that Karp strains were associated with clinically more severe human cases than Gilliam and Kato strain infections [25]. The pathogenesis of scrub typhus appears to be closely associated with the host inflammatory response–locally at the eschar, and systematically via cytokines, which play a key role in the pathology of scrub typhus [14–16,26]. High levels of several cytokines, including IL-6, IL-8, IL-10, monocyte chemoattractant protein-1 (MCP-1), macrophage inflammatory protein-1 beta (MIP-1β), IFN-γ and TNF were reported during the acute phase of scrub typhus patients, and correlated with disease severity [14,17]. Overproduction of IL-10 appears to be linked to disease severity, but in cases with low serum levels IL-10 conferred protection in patients with scrub typhus [27].

Our group previously developed mouse, cynomolgus and rhesus macaque scrub typhus models using intradermal challenge approaches and demonstrated the utility of rhesus macaques for evaluating correlates of protection in both natural and vaccine induced immunity in scrub typhus [28–30]. However, the inoculum dosages did not induce sufficiently severe pathology to perform complete assessment of vaccine-induced protection; the rhesus model using Karp strain at a dosage of 10^6^ of murine lethal dose 50 (muLD_50_) in mice, induced evident clinical disease, but not lethal infection. Despite maximising the inoculum preparation for Karp strain to a dosage of 10^7.8^ muLD_50_ in outbred mice, no further increase in disease severity was observed in rhesus macaques. Historical evidence suggests that the Gilliam strain was associated with more severe disease in both Silvered-leaf and cynomolgus macaques, we opted to use a virulent Gilliam strain-based inoculum to compare the associated symptoms to the previously characterized Karp preparations in the sense of an “inoculum bridging study”–i.e. bridging from Karp-based to Gilliam-based inocula to increase the disease severity in NHPs, in view to increase the endpoint spectrum for vaccine evaluation studies in the future.

In this study, we compared the induced pathologies of Karp versus Gilliam strains in an intradermal rhesus macaque model, using inocula previously assessed in mouse models. Standardization of inocula across both animal models and *O*. *tsutsugamushi* strains is difficult due to the variable virulence associated with hosts and the limitations in the quantitation of viable infective organisms in the applied inoculum. To optimally characterise inoculum production, we used functional endpoints; i.e. dosages inducing infection (murine ID50) and dosages inducing death (murine LD50) in outbred mice. The murine infectious dose for which 50% of susceptible mice will become infected (ID_50_) for both the Karp and the Gilliam strains are similar in the outbred scrub typhus mouse model. Hence, we applied the highest infectious doses of Karp inoculum of 10^7.8^ muID50 and 10^7.8^ muLD_50_ (the same for both functional endpoints) to the macaque model and prepared a new Gilliam inoculum of 10^8.5^ muID_50_ and 10^3.6^ muLD_50_. We hypothesized that these inocula would lead to similar NHP pathology in a comparison study, with the aim to increase dosage further according to the virulence, in order to develop a lethal NHP model.

## Materials and methods

### Ethics statement

#### NHP model

All animal research was performed strictly under approval of the Institutional Animal Care and Use Committee (IACUC) and Biosafety Review Committee at the Armed Forces Research Institute of Medical Sciences (AFRIMS) Bangkok, Thailand, an AAA-LAC International-accredited facility. The IACUC protocol number was PN15-02 (first approved February 18, 2015).

#### Mouse model

Swiss CD-1 outbred mice were used for the determination of *O*. *tsutsugamushi* inoculum titers. All animal research was performed strictly under approved IACUC (IACUC protocol #15-IDD-37; approved December 03, 2015) after approval by the IACUC and Biosafety Review Committee at the Walter Reed Army Institute of Research (WRAIR)/Naval Medical Research Center (NMRC), Silver Spring, MD 20910 USA.

The animal research was conducted in compliance with the Animal Welfare Act, and all applicable U.S. Department of Agriculture, Office of Laboratory Animal Welfare and U.S. Department of Defense guidelines. All animal experiments adhered to the principles stated in the Guide for the Care and Use of Laboratory Animals, NRC publication, 8th Edition, 2011 [31].

### Animal studies

#### NHP model

Rhesus macaques were housed individually in standard squeeze-type stainless steel cages with a minimum floor space of 4.4 square feet equipped with standard enrichments and exposed to ambient environmental conditions inside an Animal Biosafety Level 3 (ABSL-3) containment laboratory. All NHPs were fed daily with commercially prepared old-world primate extruded feed and supplemented with fresh fruit or vegetable four times per week. Fresh chlorinated water (5–10 ppm) was provided *ad libitum* via automatic water valves. Cages were cleaned daily and sanitized biweekly. Animals were trained for 2–3 weeks for pole-collar-chair restraint prior to the commencement of the study in which no anesthesia was required. All other procedures were performed under anesthesia using ketamine hydrochloride, and all efforts were made to minimize stress, improve housing conditions, and to provide enrichment opportunities.

#### Mouse model

Swiss CD-1 outbred female mice, 7–8 weeks of age were housed in an ABSL-3 facility at the WRAIR/NMRC, Silver Spring, MD 20910 USA to determine *O*. *tsutsugamushi* Karp and Gilliam strains inoculum seed titers (homogenates of 10^7^ bacteria per ml). To determine the inoculum titers, mice were i) inoculated IP with 0.2 ml suspensions of ten-fold serial dilutions (10^8^ to 10^3^) of the seed material in Snyder I buffer solution; ii) monitored daily for signs of morbidity and mortality for 21 days, and iii) were euthanized when moribund. The 50% lethal dose (LD_50_) was determined according to the methods of Reed & Muench [32] and the 50% infectious dose (ID_50_), titers were performed using ELISA to evaluate blood from survivors of the LD_50_ determinations as described previously [33].

### *O*. *tsutsugamushi* strains for inocula

The human isolate prototypes Karp (Papua New Guinea, 1943) and Gilliam (Burma, 1944) strains were cultured in 5–6 day-old chicken embryonated eggs for 7–10 days at 35°C after which the infected yolk sacs were harvested and pooled. The passage history for both NMRC strains are: *O*. *tsutsugamushi* Karp (New Guinea, Human, 1943; L9E2; yolk sack seed prepared on 4 January, 2013) and *O*. *tsutsugamushi* Gilliam (Assam-Burma Border, Human, 1944; E164 (L3) E5A4); yolk sac seed prepared on 8 March 2000). The yolk sacs were maintained in a 20% yolk sac/Snyder’s 1 buffer (w/v), homogenized and kept frozen at -80°C at the NMRC, Silver Spring, Maryland, USA until used for this study. Previously, liver-spleen homogenates containing *O*. *tsutsugamushi* Karp prototype strain was used at 10^6^ muLD_50_ for clinical time-course evaluations in our NHP scrub typhus intradermal challenge model [28,30]. As the resulting pathology was limited to mild-to-severe symptoms, an increased dosage was utilized to determine a possible lethal dose for NHPs. This led to the “Dose-increment study” (DIS) with Karp dosages infected yolk sac of 10^6^, 10^7^ and the maximum of 10^7.8^ muID_50_/muLD_50_. In the DIS study, the inocula induced sufficient signs for assessing vaccine response and treatment modalities. For the Gilliam strain, the prepared yolk sac inoculum had a muID_50_ of 10^8.5^ and a muLD_50_ of 10^3.6^. Historically, Gilliam strains have been associated with more severe disease than Karp strains in developing scrub typhus in NHPs (19). Thus, we were confident in using this inoculum for the evaluation of NHP disease presentations in this study.

In addition to determining the muID_50_ and muLD_50_ for the 0.2 ml infective yolk sac inocula, they were quantitated for *O*. *tsutsugamushi* bacterial loads by staining (Invitrogen Live/Dead BacLight Bacterial Viability Kits, Thermo Fisher Scientific, Waltham, MA) and the Otsu47 quantitative real-time PCR (qPCR) assay [34]. For the Karp strain the number of live bacteria was determined to be 1.35x10^9^/0.2 ml and number of genome copies was 3.8x10^8^/0.2 ml. For the Gilliam strain the number of genome copies was determined to be 4.4x10^9^/0.2 ml. In this study we applied 200 μl of Karp inoculum at dose 10^7.8^ muID_50_ and 200 μl of Gilliam at dose 10^8.5^ muID_50_ administered via the intradermal route to the NHPs as described previously [28,30] and in S1 Table.

### Non-Human Primate (NHP) scrub typhus model

**Fig 1:**
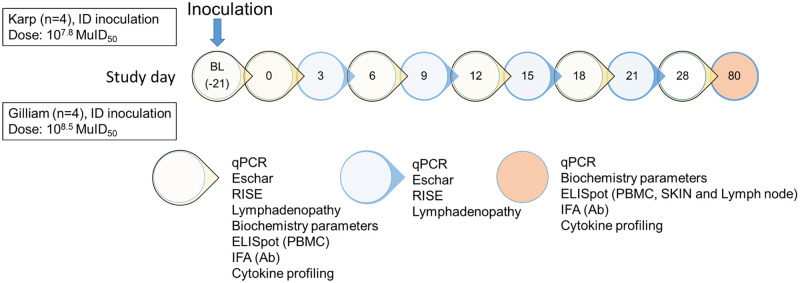
Schematic representation of the experiment schedule of this study. At day 0, all macaques were inoculated intradermally with either *O*. *tsutsugamushi* strain Karp (n = 4) or Gilliam (n = 4). The laboratory assays performed at the designated time point are indicated. BL: Baseline 21 days prior inoculation, MuID_50_ = Murine infectious dose that infects 50% of mice inoculated. ID = intradermal.

Our group previously characterized the rhesus macaque (*Macaca mulatta*) as a scrub typhus model using an intradermal challenge with *O*. *tsutsugamushi* Karp at AFRIMS [28]. The same model served for investigations in this study; a total of eight Indian-origin rhesus macaques (*Macaca mulatta*, 2 male and 6 female) were randomly assigned using SPSS for intradermal inoculation with either *O*. *tsutsugamushi* strain Karp (n = 4, 1 male and 3 female) or Gilliam (n = 4, 1 male and 3 female) on the anterior thighs (2 sites of inoculation of each animal, each on the left and right thigh). The study design incorporated a within-subject approach, whereby every animal served as its own control by providing baseline samples prior to inoculation. The animals were raised at AFRIMS since birth, ranged from 7 to 15 years of age, weighed between 8.6 to 11.4 kg at the start of the study, were documented negative for Simian immunodeficiency virus (SIV), Simian type D retrovirus (SRV), Simian T-lymphotropic virus-1 (STLV-1) and herpes B virus, had no prior *O*. *tsutsugamushi* exposure according to their medical (experimental) history and were confirmed anti *O*. *tsutsugamushi*-antibody negative by indirect immunofluorescence assay (IFA). Their clinical status was monitored three times per day for adequate activity, food intake, and included daily monitoring for body temperature, development of eschar lesions at the site of inoculation and lymphadenopathy. Body temperatures were recorded daily for 7 days prior to inoculation until 28 dpi. Eschar development and eschar severity were documented using the RISE scoring system; based on the criteria Redness (erythema), Induration of the skin (infiltration), Swelling (edema), Eschar formation (necrosis), and the diameters of the lesions were documented in mm (S2 Table). Lymphadenopathy and bacteremia were quantitated by qPCR (see below) and documented every 2 days. The time-course of hematology, biochemistry and immunological findings (consisting of IFA, ELISpot and cytokine profiling) were determined at baseline (day -21) and day 0, 6, 12, 18, and 28 (dpi). In addition, extra samples (blood, skin and lymph node) were collected at 80 dpi in 3 out of 4 animals of each group for biochemistry and immunological assays. The designated experiment schedule is shown in Fig 1.

Upon completion of the study procedures (day 28), all animals were treated with 5 mg/kg doxycycline given orally twice daily for 10 days as standard treatment. No organs were collected for immunohistochemical staining or bacterial load quantitation. The macaques were returned safely to the colony after serial qPCR assays documented the absence of *O*. *tsutsugamushi* in their blood for the duration of 4 weeks.

### Clinical pathology

Blood samples were collected into lithium heparin blood collection tubes and analyzed for 16 blood biochemistry parameters using a Roche, Cobas C311 Analyzer. The panel of 16 plasma biochemistry parameters consisted of CRP, lactate, albumin, total protein, alkaline phosphatase (ALP), aspartate aminotransferase (AST), alanine aminotransferase (ALT), creatine phosphokinase (CPK), blood urea nitrogen (BUN), creatinine (CRE), total bilirubin (TBIL), total cholesterol (TCHO), calcium (CA), potassium (K), sodium (Na) and chloride (Cl).

### Molecular and serological assays

The *O*. *tsutsugamushi-*specific qPCR assay targeting the *O*. *tsutsugamushi* 47-kDa *htrA* gene, was used to determine the bacterial load from DNA extracted from 200 μl of EDTA whole blood as described previously [34]. DNA extraction was performed by using a DNeasy Blood and Tissue kit, (Qiagen, Valencia, CA, USA). The qPCR assay was performed as described using a CFX96 Real Time PCR detection system (BioRad, Hercules, CA, USA). A serial dilution of Plasmid DNA, pGEM-47kda served as standards (10^6^ to 1 copies per μl), and a confirmed Karp-positive sample from *O*. *tsutsugamushi* infected L929 (mouse fibroblast cell line) cell culture as positive control.

Anti-*O*. *tsutsugamushi* specific antibodies (IgM and IgG) were determined in serum samples by IFA as described previously [28]. Briefly, antigen coated slides with a mixture of three *O*. *tsutsugamushi* strains, Karp, Kato and Gilliam, were purchased from the Australian Rickettsial Reference Laboratory (Geelong, Australia). A two-fold serial dilution of serum samples (from 1:100 to 1: 25,600) in 2% skimmed milk PBS buffer was incubated onto the antigen slides for 30 min. After washing with PBS the slides were incubated with FITC-conjugated goat anti-monkey IgM or IgG (Brookwood Biomedical, Birmingham, AL) for 30 min, and mounted with fluorescence mounting medium (Dako, Glostrup, Denmark) for evaluation under fluorescence microscope.

### *Ex-vivo* interferon-gamma (IFN-γ) enzyme-linked immunospot (ELISpot) assay

To assess the kinetics and magnitude of the *O*. *tsutsugamushi-*specific cellular responses, the *ex-vivo* IFN-γ ELISpot assay was performed following an 18-hour stimulation of freshly isolated peripheral blood mononuclear cells (PBMC) at each designated time point as described previously [29]. Briefly, fresh PBMC were added in duplicate wells at 2x10^5^ PBMC in 50 μl per well and 50 μl of *O*. *tsutsugamushi*-specific antigen was added. In this study, a panel of four types of *O*. *tsutsugamushi-*specific antigens were used: heat-inactivated whole cell antigens (WCA) obtained from (1) Karp and (2) Gilliam strains cultured in L929 mouse fibroblasts, and (3) 56-kDa TSA and (4) 47-kDa HtrA proteins of *O*. *tsutsugamushi* Karp-like strain UT76 (the predominant circulating strain of *O*. *tsutsugamushi* in Northern Thailand) in pools of custom synthesized 20-mer peptides overlapping by 10 amino acids obtained as Pepsets peptide libraries (Mimotopes Pty Ltd, USA), derived from the full open reading frame (ORF) available on GenBank. The gene sequence similarity of the 56-kDa protein of Karp and Gilliam compared with UT76 is 87.45 and 79.59% respectively (analyzed by blastp, NCBI tool), while the 47-kDa HtrA shows high level conservation with >98% sequence similarity among the 3 strains. After 18 hours, secreted IFN-γ was detected by adding 1 μg per ml biotinylated mAb 7-B6-1-biotin for IFN-γ, which recognizes an epitope completely conserved between human and macaques in the helical region of human IFN-γ, (Mabtech AB, Sweden) for 3 hours and followed by 1 μg per ml streptavidin alkaline phosphatase (Mabtech AB, Sweden). The plates were developed, scanned (CTL ELISpot reader, Cellular Technology Limited, USA), spots counted (Immunospot 3.1 software, using the manufacturer’s automated SmartCount settings) and results were expressed as IFN-γ spot-forming cells (SFC) per million PBMC. Background responses in unstimulated control wells were always less than 20 spots/10^6^ PBMC, and were subtracted from those measured in the antigen stimulated wells.

### Specific cellular immune responses to *O*. *tsutsugamushi* antigens in blood, skin and lymph nodes

PBMC were obtained at 7 time points; baseline (-21), 0, 6, 12, 18, 28 and 80 dpi, while skin and lymph node samples serving cell-mediated immune response characterization were collected at 80 dpi only. T-cell responses in cells isolated from peripheral blood, skin and inguinal lymph node were determined by the *ex-vivo* IFN-γ ELISpot assay at day 80 dpi (2×10^5^ cells per well in duplicate). Skin shaving specimens enabled isolation of cells from the epidermis and dermis layers using the following methodology; skin tissue was cut into small pieces and incubated in RMPI + 10% FBS containing 1 mg per ml Dispase (Invitrogen GIBCO, USA) for 2 hours in 37°C CO_2_ incubator to separate the epidermis and dermis layer. Next, the separated epidermis and dermis layer were incubated in RPMI + 10% FBS containing 0.8 mg per ml collagenase type IV (Worthington-Biochemical Corp, NJ, USA) for 2 hours in 37°C CO_2_ incubator before passing through a 70 μm strainer to obtain a single cell suspension. For single cell isolation from lymph node, lymph node was cut into small pieces and undergone 2 treatments with the enzymes similar with the skin tissue before passing through a 70 μm strainer to obtain a single cell suspension.

### Cytokine profile determinations by Multiplex assay

50 μl of serum samples from the infected macaques were analyzed in duplicate for 23 immune mediators using the Milliplex MAP Non-Human Primate Cytokine Magnetic Bead Panel-Immunology Multiplex assay (PRCYTOMAG-40K, Milliplex, Merck, USA) and analyzed by Bio-Plex 200 system (BioRad, USA) according to the manufacturer’s instruction. The 23 cytokines consisted of G-CSF, granulocyte-macrophage colony-stimulating factor (GM-CSF), IFN-γ, IL-1ra, IL-1β, IL-2, IL-4, IL-5, IL-6, IL-8, IL-10, IL-12/23 (p40), IL-13, IL-15, IL-17, IL-18, MCP-1, MIP-1α, MIP-1β, sCD40L, transforming growth factor alpha (TGF-α), TNF and vascular endothelial growth factor (VEGF). The final concentrations of the analyses were calculated using MILLIPLEX v5.1 Analyst software. Due to a technical problem majority of sCD40L concentrations were out of limit and were omitted from the analysis.

### Statistical analysis

Statistical analysis of differences and correlations in bacteremia, cellular immune responses, humoral responses, immune mediators, and ratios were performed using GraphPad Prism 7 (GraphPad Software Inc., La Jolla, CA, USA). The results between the Karp versus Gilliam infected groups are expressed as medians with interquartile range (unless otherwise specified) and were compared using the non-parametric Mann-Whitney t-test for two comparisons or the Wilcoxon test for paired analysis. Significant differences between individual time points within a group were determined with the non-parametric Kruksal-Wallis one way ANOVA and Dunn’s multiple comparisons test. The relationship between each set of values were evaluated using Spearman’s rank correlation test. Two-tailed P values < 0.05 were considered statistically significant. Random assignment of the experiment group was performed in SPSS software version 22.0 (IBM Corp, NY, USA).

## Results

### Temperature and bacteremia

**Fig 2:**
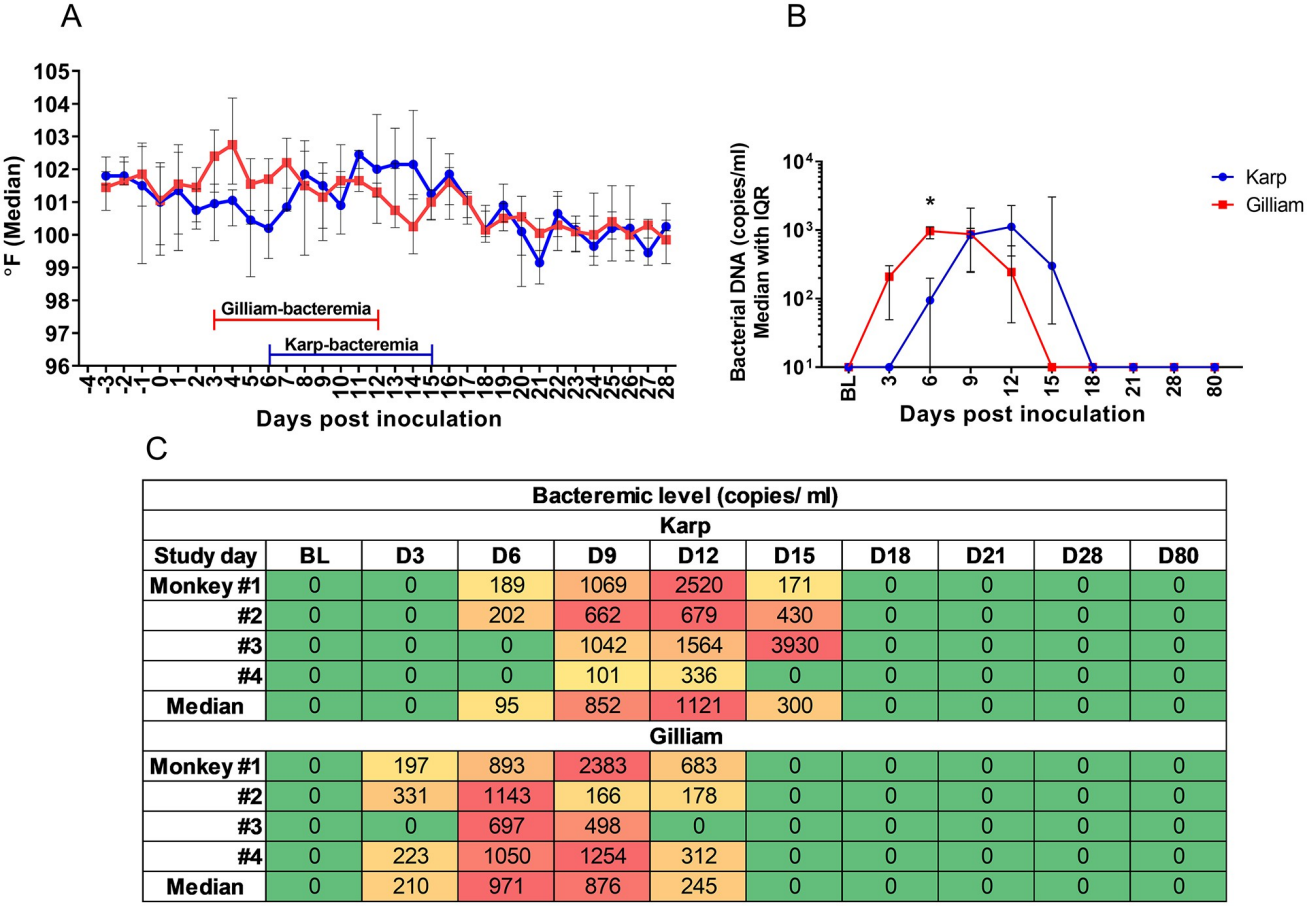
Daily body temperature charts ranging from 0 to 28 days post infection (dpi) following inoculation with *O*. *tsutsugamushi* strain Karp, n = 4 (red line) and Gilliam, n = 4 (blue line). Temperatures (°F) are represented as median lines with IQR (A). The kinetics of bacteremia (B) and the heat map of individual bacteremia levels (C) for the Karp and Gilliam infected macaques between 0 to 80 dpi (median with IQR) are shown. In the bacteremia heat map of bacterial load (copies/ml), green indicates a negative PCR result, yellow 1–500 copies/ml, orange 500–1,000 copies/ml and red indicates >1,000 copies/ml. Comparison of bacteremia levels between Karp and Gilliam infected animals was performed using the Mann Whitney t-test, p value < 0.05 denoting significance (*).

Following intradermal inoculation, the Gilliam-inoculated animals developed an earlier onset of febrile temperatures (starting from 3 to 15 dpi), but similar duration of elevated body temperatures and bacteremia compared to the Karp-inoculated animals (starting from 6 to 18 dpi), respectively (Fig 2A and 2B). A paradoxical drop in body temperature preceded the onset of bacteremia as also observed in the previous NHP study [28,30]. The median body temperature during the bacteremic phase, was significantly higher than that of the post-bacteremic phase. In Karp-infected macaques the median body temperatures were 101.8 °F during the bacteremic phase and 100.3 °F for the post bacteremic phase (p = 0.0005). In Gilliam-infected macaques the median body temperatures were 101.5 °F during the bacteremic phase and 100.3 °F for the post bacteremic phase (p < 0.0001).

Mean time to onset of bacteremia in the Gilliam infected macaques was shorter by 3 days compared to the Karp group. The Gilliam-bacteremia started to be detectable at 3 dpi in the majority of the animals (3 out of 4 animals), then reached the mean peak level at 9 dpi (1,075 copies/ml; range: 166–2,383; %CV 91.5) and was undetectable at 15 dpi (Table 1). However, in the Karp group, bacteremia became detectable at 6 dpi in 2 out of 4 animals followed by the mean peak (1,274 copies/ml; range: 679–2,520; %CV 76.7) at 12 dpi, and was undetectable at 18 dpi. The mean bacteremic phases of the both groups were similar, with a mean of 9 days in duration (range 3 to 9 days) as shown in Fig 2B and 2C. A higher coefficient of variance for bacteremia was observed in the Gilliam-infected macaques. A significant difference of bacterial loads between the two groups was observed only at 6 dpi at the onset of Karp-bacteremia. There was no significant difference in bacterial loads for the mean peak levels (p = 0.8858) (Fig 2).

**Table 1:** Ratios of median cytokine to IL-10 serum levels for Karp / Gilliam strain infected macaques from baseline to 80 dpi. Comparisons of ratios between the two groups with a statistically significant difference are depicted with an asterisk (Mann Whitney t-test, N/A = not available, as cytokine values were below assay detection limit).

Ratios	Median of cytokine and acute inflammation ratios: Karp/Gilliam					
	BL	Day 6	Day 12	Day 18	Day 28	Day 80
**IL-6/IL-10**	0/0	0/0.36	0.19/0.21	0/0	0.06/(N/A)	(N/A)/0
**TNF/IL-10**	1.21/2.96	0.83/0.18	0.37/0.67	0.95/0.92	1.20/(N/A)	(N/A)/1.47
**IFN-**γ**/IL-10**	0/0	0.26/6.86 (*)	0.14/0.25	0/0	0/(N/A)	(N/A)/0
**CRP/IL-10**	0.50/0.84	0.08/1.91 (*)	0.31/0.49	0.06/0.05	0.17/0	(N/A)/1.13

### Eschar lesion development and lymphadenopathy

**Fig 3:**
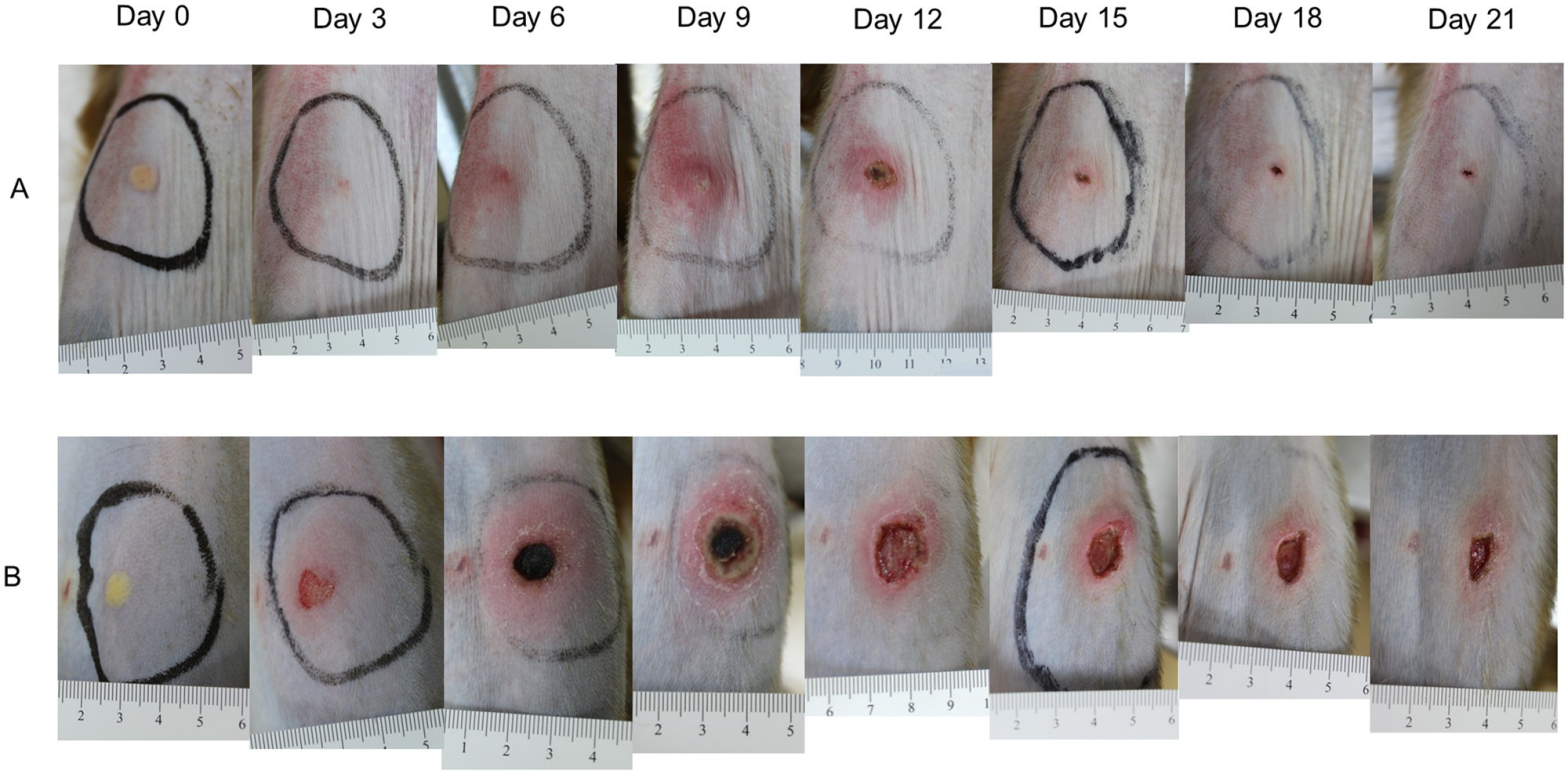
Time-course development from 0 to 21 dpi of the inoculation eschar at the anterior thigh after intradermal inoculation with *O*. *tsutsugamushi* strain Karp (Panel A) and Gilliam (Panel B). A centimeter scale is shown below each lesion at the day of observation.

**Fig 4:**
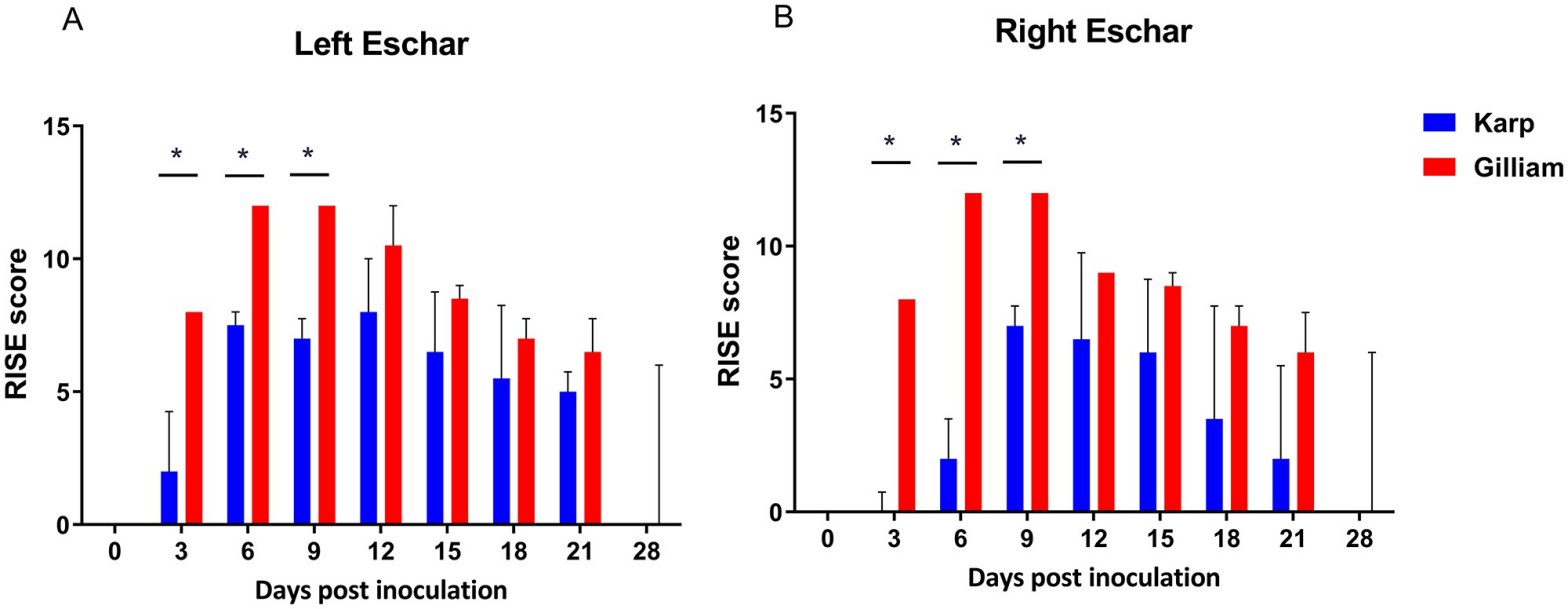
Time-course evaluation of eschar severity between Karp (n = 4) and Gilliam (n = 4) inoculated macaques as determined by the RISE score (maximum score = 12, score details in S2 Table). Eschars on the left (A) and right (B) thighs showed greater severity for Gilliam-infected macaques at 3, 6, 9 dpi. A significant difference (p value < 0.05) in RISE scores is marked with an asterix (*); analyzed by Mann Whitney t-test with two-tails.

Animals were monitored every other day between 0 to 28 dpi. Following ID inoculation, earlier formation of more pronounced and slower healing eschar lesions were observed in the Gilliam group (Fig 3). The individual criteria for the RISE lesion severity score were determined and summarized over time (Fig 4 and S2 Table). At the inoculation site of Gilliam-infected macaques, a small erythematous and indurated plaque developed at 3 dpi, whereas these were observed at 6 dpi in the Karp-infected group–co-inciding with bacteremia onset. Generally, the eschar lesions in all Gilliam-infected macaques were larger, with raised borders and unequivocal central necrosis on day 6. The lesions were most pronounced on day 9 with 14–20 mm necrotic centers (mean 15.75 mm, SD ± 1.91) and perifocal erythema of 24–40 mm (mean 29.75 mm, SD ± 5.63) in diameter. The lesions of the Gilliam infected animals healed by 28 dpi, except for one where the eschar crust (8 mm diameter) remained until 28 dpi.

The eschar lesions in the Karp group were less severe compared to the Gilliam group, one Karp-infected macaque did not develop a typical black crust eschar lesion. At 12 dpi, the eschar lesion in the Karp animals peaked with 7–10 mm necrotic crusts (mean 6.38 mm, SD ± 4.07) and erythema of 10–25 mm (mean 17 mm, SD ± 5.56) in diameter (Fig 3). The lesions healed by 28 days in all macaques of the Karp group, and RISE severity scores are summarized in S2 Table, time course data are shown in Fig 4. Lesion severity comparison of Gilliam and Karp group using RISE system found significantly higher scores in the Gilliam group at 3, 6 and 9 dpi on the both sites of the inoculations (p = 0.0286 at 3, 6 and 9 dpi).

### Formation of lymphadenopathy

**Fig 5:**
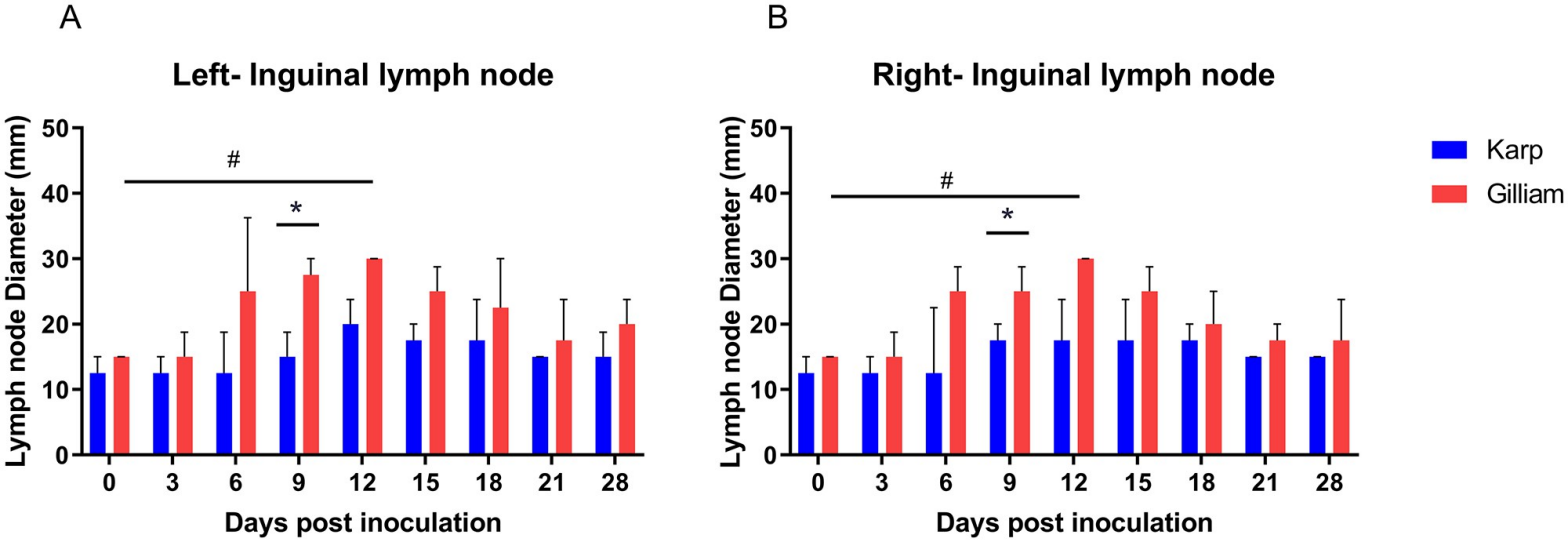
Development of lymphadenopathy of ID inoculated macaques with *O*. *tsutsugamushi* strain Karp (n = 4) and Gilliam (n = 4). Diameter measurements of left (A) and right (B) inguinal lymph nodes between 0 and 28 dpi are shown. Statistical differences (p values <0.05, Mann Whitney t-test) are marked with an asterix (*). The differences between individual time points were analyzed by One Way ANOVA (Kruskal Wallis and multiple comparisons (Dunn’s), # denotes a p value <0.05.

Two sites were monitored; inguinal and axillary draining lymph nodes. All animals developed lymphadenopathy. More pronounced findings were observed in the Gilliam group, with significant enlargement of inguinal lymph nodes compared to Karp seen at 9 dpi bilaterally (p = 0.0286, Fig 5). Within Gilliam infected animals, significantly enlarged lymph nodes compared to baseline were seen at day 12. Over the study course, no significant enlargement of axillary lymph nodes was found (Fig 5).

### Time-course of biochemical markers and correlations with the bacteremia

**Fig 6:**
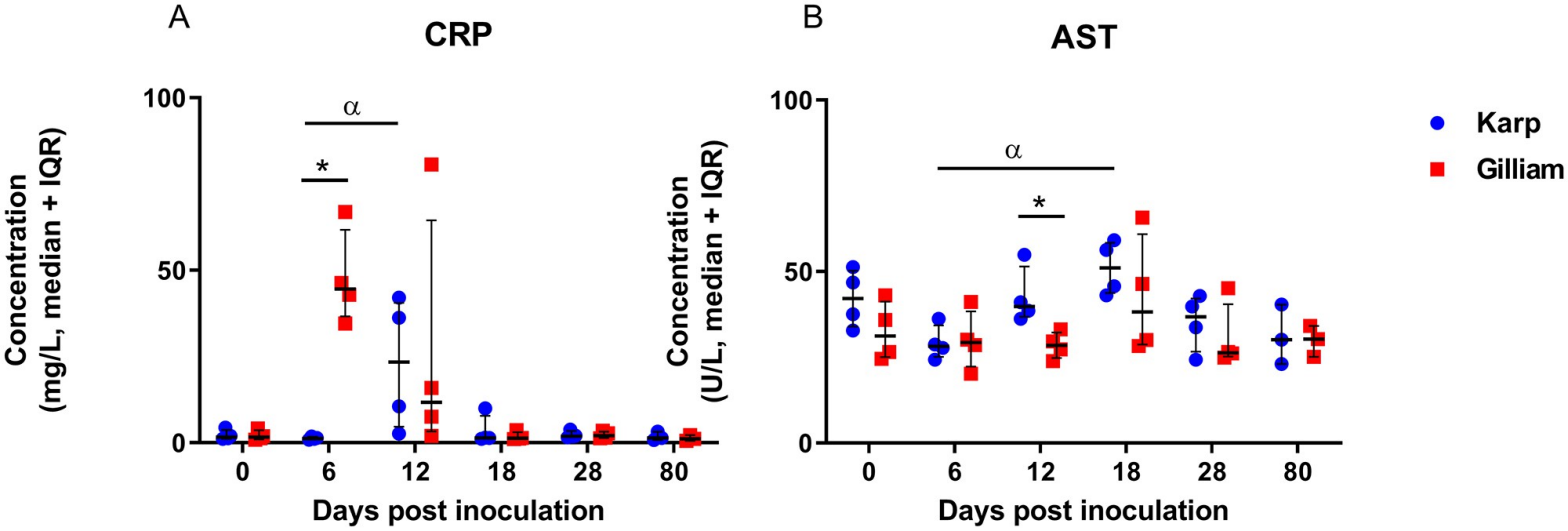
Scatter plots for CRP (mg/L) and AST (U/L) plasma concentrations for Karp (n = 4) and Gilliam (n = 4) infected macaques. Time-course data from 0 to 80 dpi, statistically significant differences at specific time points are indicated with an asterisk (p value <0.05, Mann Whitney t-test), and with α for differences between time points (p< 0.05, Kruskal-Wallis ANOVA test with Dunn’s multiple comparisons). Additional biochemistry data are presented in S2 Table.

**Fig 7:**
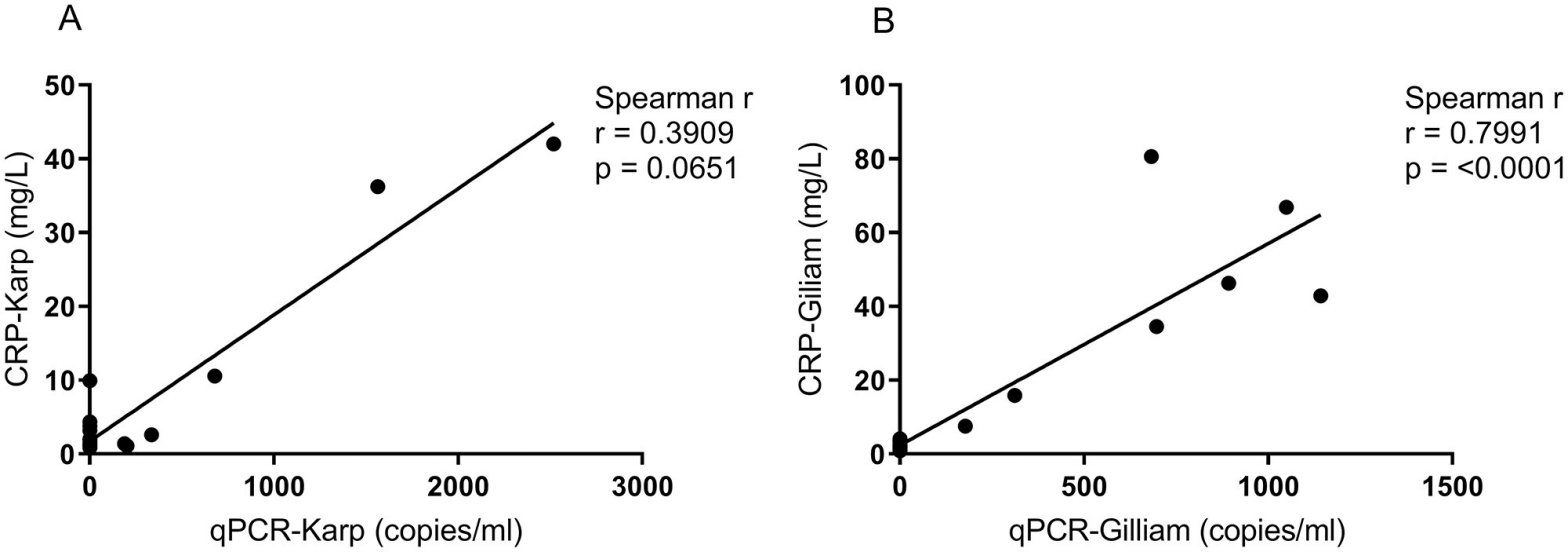
Correlation between CRP plasma levels (C311 Automated Chemistry Analyzer) and bacterial load (qPCR, copies/ml) for Karp, n = 4 (A) and Gilliam, n = 4 (B) strain *O*. *tsutsugamushi* infections in macaques.

A panel of sixteen plasma biochemistry values were analyzed (S3 Table). In this study, blood biochemistry values associated with disease progression included: CRP (elevated during bacteremia); lactate, AST and ALP (peaked at 18 dpi); and TBIL (highest at 6–12 dpi). Biochemistry profiles and kinetics of the biochemistry values were similar between the two groups, except for CRP (significantly higher at 6 dpi for Gilliam, p = 0.0286) and AST (significantly higher at 12 dpi for Karp, p = 0.0286) (Fig 6). Compared to the baseline, CRP levels were markedly increased during the peak of the bacteremia; for the Gilliam group at 6 dpi, while for Karp at 12 dpi (Fig 6A). Generally, AST levels were not significantly increased in NHPs for both Karp and Gilliam strains. AST levels were slightly higher in the Karp-infected macaques compared to the Gilliam- infected macaques, although these were significantly higher than Gilliam-infected macaques at 12 dpi only, and within the Karp group levels at 12 dpi were significantly higher than at 6 dpi (Fig 6B). As CRP—an acute phase-secreted protein—was elevated during bacteremia, the correlation with bacterial load was calculated. A significant positive correlation between CRP levels and bacterial load was found only in Gilliam-infected macaques, Spearman’s r was 0.7991(p < 0.0001) (Fig 7A and 7B).

### Kinetics of the anti-*O*. *tsutsugamushi* antibody responses

**Fig 8:**
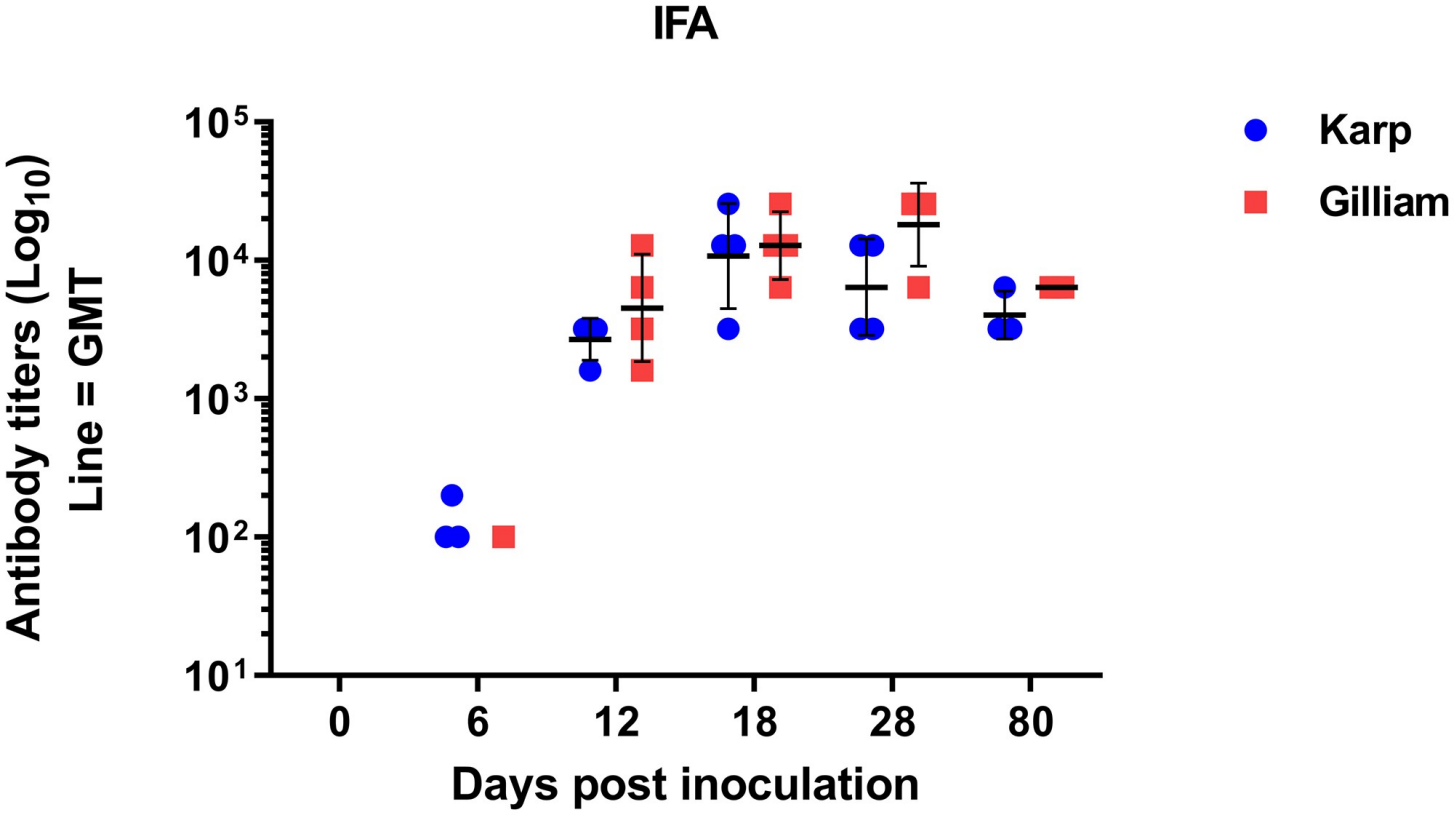
Scatter plot of antibody titers (Log_10_ scale, geometric mean with SD) representing the anti-*O*. *tsutsugamushi* specific antibody response kinetics. Data are combined IgM + IgG levels, against whole cell *O*. *tsutsugamushi* antigen. No statistical differences were found between both groups at all time points (Mann Whitney t-test). Antibody titers are presented in S3 Table.

Seroconversion of anti-*O*. *tsutsugamushi* antibodies (combined IgM+IgG) was observed at 6 dpi in 3 of 4 Karp-infected and one of 4 Gilliam-infected macaques (Fig 8). At 12 dpi, anti*-O*. *tsutsugamushi* antibody responses were detectable in all macaques in both groups. The peak antibody responses were observed following bacteremia clearance at 18 dpi in the Karp-infected macaques and at 28 dpi in the Gilliam infected macaques. One of the Karp-infected animals and 3 of 4 Gilliam-infected macaques reached the maximum antibody titer of 1:25,600 at 28 dpi, however, no statistical differences were observed at any time point when the mean geometric titers between both groups were compared (Mann Whitney t-test, S4 Table). At 80 dpi, the specific antibodies started to decline and were still detectable in all animals in both groups; the geometric mean overall titers were 1:4,031 and 1:6,400 for Karp- and Gilliam-infected macaques, respectively. In conclusion, the Karp-infected macaques showed a shorter time-to seroconversion but tended to have lower antibody geometric mean titers in this study (no statistical significance).

### Cytokine and chemokine mediated responses and correlations with bacteremia

**Fig 9:**
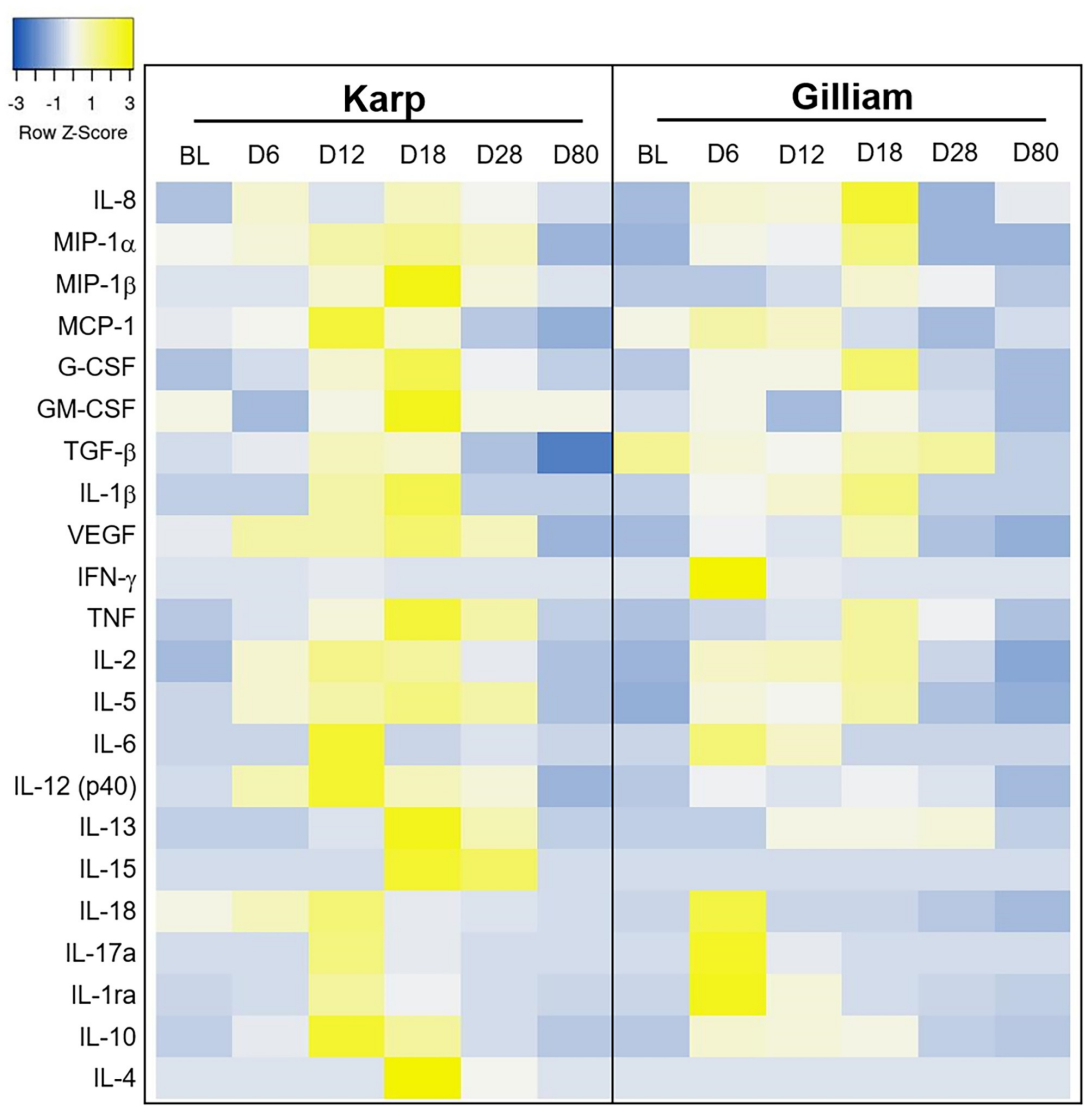
Time-course dynamics of the serum immune mediators in a heat map (heatmapper, http://www.heatmapper.ca/expression/). Each row represents a mean cytokine serum level per time point (baseline BL, Day 6, 12, 18, 28 and 80). The color represents the strength of expression levels (blue low and yellow high). Median concentrations of all individual immune mediators are summarised in S5 Table.

Multiple cytokines, chemokines and growth factors were up-regulated, and the immune mediator expression patterns were similar for both groups in relation to their bacteremia and temperature curves (Fig 9). Of note, IFN-γ, IL-1ra and IL-15 cytokine serum levels were significantly raised in the Gilliam group at 6 dpi, when compared to the Karp group (Mann Whitney t-test, p = 0.0286) for all cytokines.

The heat map of the immune mediator expression levels demonstrated that in both groups, pro-(IFN-γ, IL-15, IL-6, IL-17a and IL-18), anti-(IL-1ra and IL-10) inflammatory cytokines, and chemokine (MCP-1) reached the highest levels during peak bacteremia (Fig 9); for the Gilliam bacteremia (3 to 12 dpi, peak at 6 dpi), and for Karp bacteremia (6 to 15 dpi, peak at 12 dpi), respectively. Peak serum levels of MIP-1a, MIP-β, G-CSF, VEGF, TNF, IL-2 and IL-12 (p40) were observed after the bacteremia was controlled for both groups. The majority of the immune mediators returned to the baseline levels by 28 dpi and were almost undetectable at 80 dpi (S5 Table).

**Fig 10:**
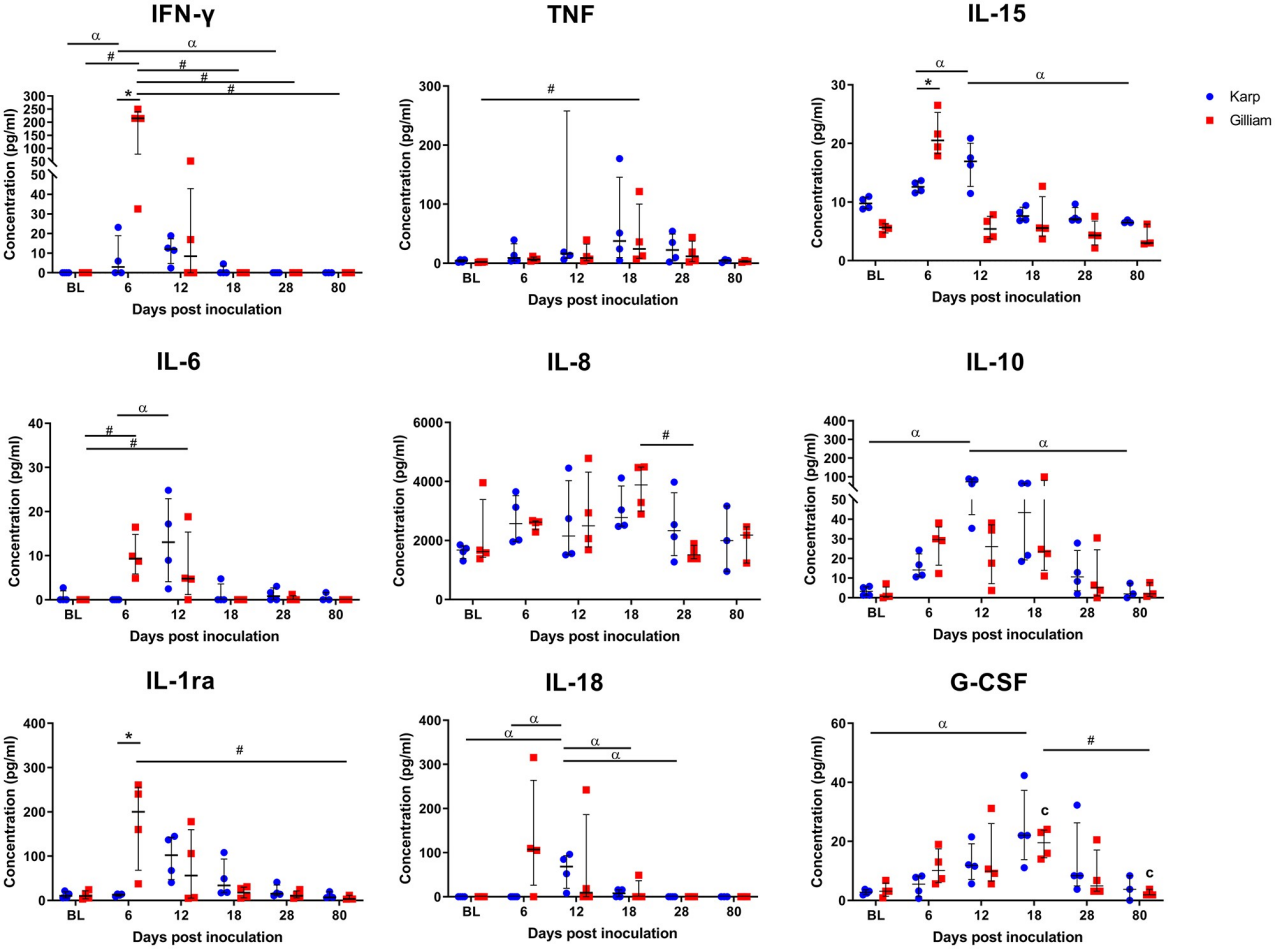
Scatter dot plots of serum cytokine levels for Karp (blue) and Gilliam (red). Pronounced differences were seen for the nine cytokines depicted in this figure. Statistically significant differences at one time point are indicated with an asterisk (p value <0.05, Mann Whitney t-test)–for IFN-γ, IL-15 and IL-1ra cytokines. Statistically significant differences between time points are indicated with α for Karp strain values and # for Gilliam strain values (p <0.05, Kruskal-wallis one way ANOVA followed by Dunn’s multiple comparisons).

Comparisons between Karp and Gilliam-infected macaques at individual time points revealed that nine cytokines reached significantly different levels between the groups (One-way ANOVA Kruskal Wallis followed by Dunn’s multiple comparisons test, Fig 10); IFN-γ (Karp; BL vs 12 dpi (p = 0.0488), 12 vs 28 dpi (p = 0.0488) and Gilliam; BL vs 6 dpi (p = 0.0142), 6 vs 18 dpi (p = 0.0142), 6 vs 28 dpi (p = 0.0142), 6 vs 80 dpi (p = 0.0331)), TNF (only in Gilliam at BL vs 18 dpi (p = 0.0237, IL-6 (Karp; at 6 vs 12 dpi (p = 0.0254) and Gilliam; at BL vs 6 dpi (p = 0.0361), 6 vs 18 dpi (p = 0.0361)), IL-8 (only in Gilliam at 6 vs 18 dpi (p = 0.0315)), IL-15 (only in Karp; at 6 vs 80 dpi (p = 0.0458), 12 vs 80 dpi (p = 0.0122)), IL-18 (only in Karp; at BL vs 6 dpi (p = 0.0179), 6 vs 12 dpi (p = 0.0179), 12 vs 28 dpi (p = 0.0179), 12 vs 80 dpi (p = 0.0405) IL-1ra (only in Gilliam at 6 vs 80 dpi (p = 0.0317)), IL-10 (only in Karp at BL vs 12 (p = 0.126), 12 vs 80 dpi (p = 0.0299)) and G-CSF (Karp at BL vs 18 dpi (p = 0.0258) and Gilliam at 18 vs 80 dpi (p = 0.0497)).

**Fig 11:**
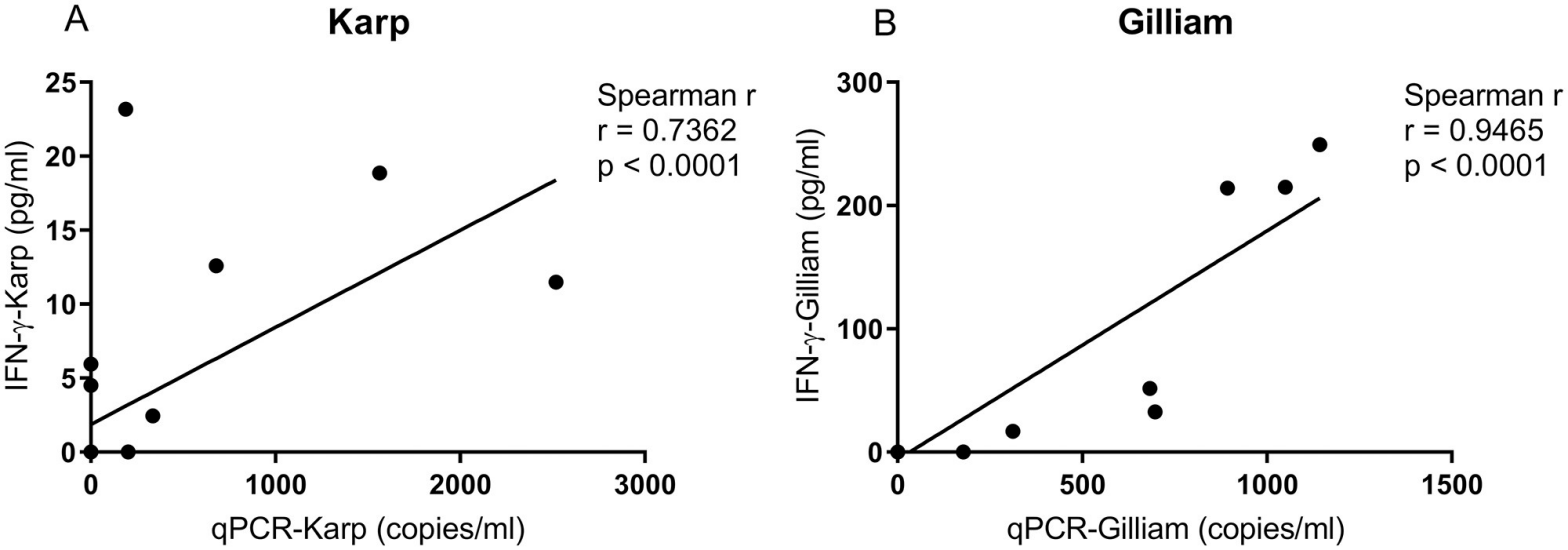
Correlations of IFN-γ cytokine serum levels (ρg/ml) with bacterial load (qPCR, copies/ml) for Karp, n = 4 (A) and Gilliam, n = 4 (B) strain infected macaques. Spearman’s correlation revealed a stronger correlation for IFN-γ with bacterial load in Gilliam strain infected macaques, Spearman r correlation = 0.9465 (p<0.0001).

A strong positive correlation between IFN-γ serum levels and bacterial load (47 kDa *htra* gene copy number/ml) was found for both groups; for Karp- (Spearman r = 0.7632, p < 0.0001), and for Gilliam-inoculated macaques (Spearman r = 0.9465, p < 0.0001), respectively. Confidence intervals and goodness of fit was improved for the Gilliam-inoculated animals (Fig 11).

### Ratios of pro-inflammatory and anti-inflammatory cytokine levels

The balance between pro- and anti-inflammatory cytokine levels is important in controlling infection for several human diseases; the ratio of pro- to anti-inflammatory cytokines characterizes Th1- or Th2 dominant responses. We calculated the ratios of IL-6, TNF, IFN-γ and CRP levels independently to IL-10 levels. Comparisons between the Karp- and Gilliam-infected macaques were analyzed by Mann Whitney t-test (Table 1). At 6 dpi, the IFN-γ/IL-10 and CRP/IL-10 ratios in the Gilliam group were significantly higher than in the Karp-infected macaques (IFN-γ/IL-10 median ratio in the Karp = 0.26 (95%CI -0.56–1.51) versus Gilliam group = 6.89 (95%CI 2.39–9.47) at p = 0.0286) and CRP/IL-10 (median ratio in the Karp group = 0.08 (95%CI 0.037–0.184) versus Gilliam group = 1.91 (95%CI 0.98–3.43) at p = 0.0286).

### Specific cellular immune responses to *O*. *tsutsugamushi* antigens in blood, skin and lymph nodes

#### Whole cell antigen-*O*. *tsutsugamushi* Karp (WCA-OT)

**Fig 12:**
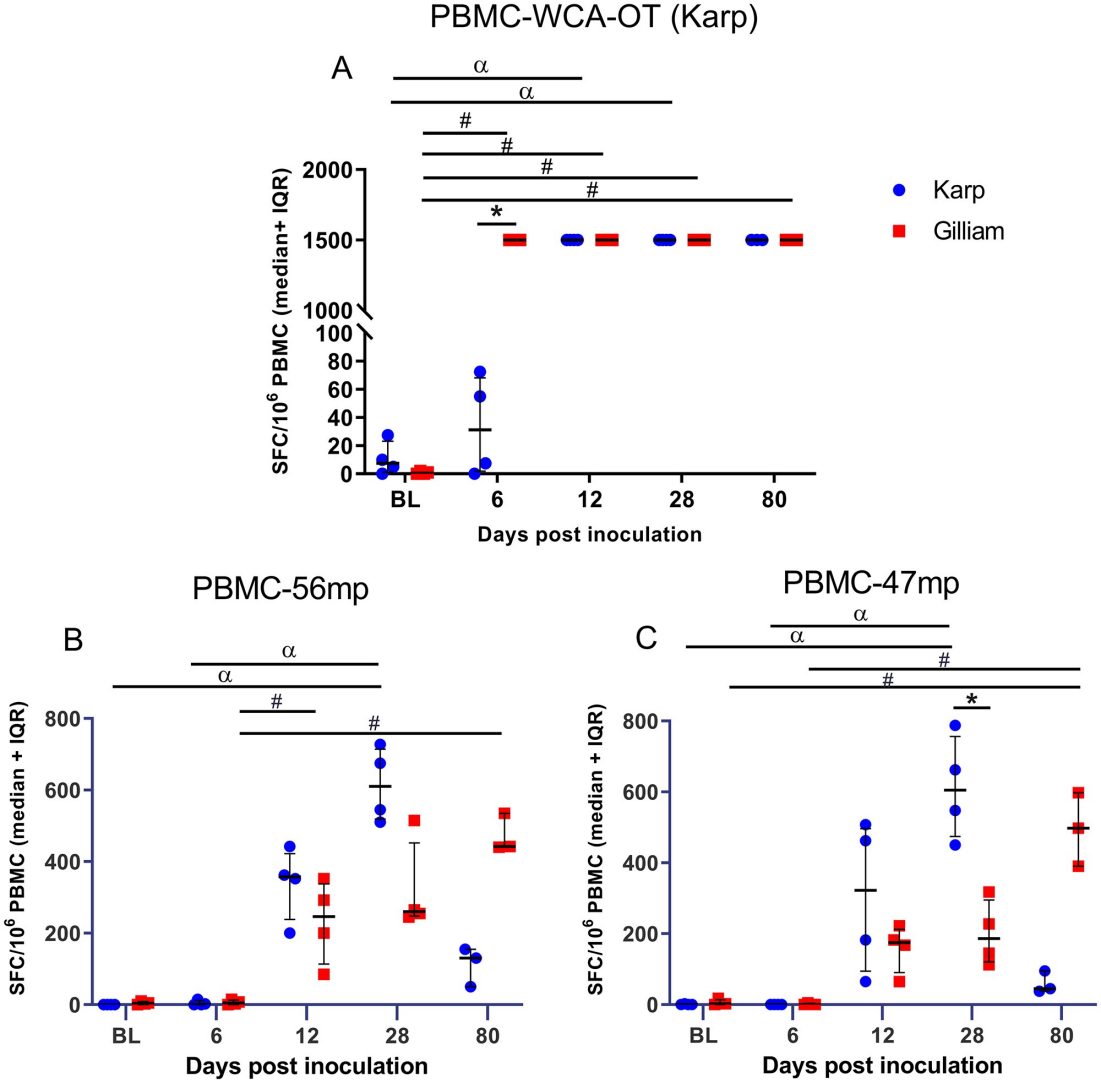
Frequency of antigen-specific IFN-γ secreting cells following stimulation of PBMCs with whole cell antigen *Orientia tsutsugamushi* (WCA-OT) antigen (Karp, A), 56- kDa peptide megapool (B) and 47-kDa peptide megapool (C). Data are medians and 25–75 percentiles of spot forming cells per 10^6^ PBMCs for Karp (blue) and Gilliam (red) infected macaques. Time-course data ranging from baseline (BL) at day -21, to 28 and 80 days post infection (dpi). Statistically significant differences in the magnitude of cellular responses for both groups at one time point are indicated with an asterisk (p value <0.05, Mann Whitney t-test), and between time points are indicated with α for Karp strain values and # for Gilliam strain values (Kruskal-Wallis one way ANOVA followed by Dunn’s multiple comparisons).

The magnitudes of IFN-γ-secreting cell responses against WCA-OT were significantly increased for both groups. The Karp-infected NHPs reached maximal levels at 12 dpi, and the Gilliam-infected NHPs at 6 dpi–at 6 dpi the difference between Karp and Gilliam NHPs was significant (p = 0.0286) (Fig 12A). IFN-γ producing cells for the Gilliam group remained high until 80 dpi, while those from the Karp group declined over time. Significant increases of the specific cellular immune response to WCA-OT (Karp) was found between baseline and all time points for Gilliam infected macaques, while for the Karp-infected macaques significant rises were seen at 12 and 28 dpi only. Corresponding data for IFN-γ-secreting cell responses against WCA-OT strain Gilliam could not be obtained due to technical limitations in antigen preparation.

#### 56-kDa and 47-kDa-peptide megapools (56mp and 47mp)

The specific cellular responses to 56mp and 47mp are shown in Fig 12B and 12C, respectively. The strongest specific cellular responses to 56mp and 47mp from Karp-infected macaques were found at 28 dpi, while in Gilliam-infected macaques the development was more gradual with the peak at 80 dpi. When compared the specific cellular responses to 56mp between individual time points there were significant differences between BL vs 28 dpi (p = 0.0286) and 6 vs 28 dpi (p = 0.0286) for Karp-infected macaques and between 6 vs 12 dpi (p = 0.0286), 6 vs 28 dpi (p = 0.0286) and 6 vs 80 dpi (p = 0.0286) for Gilliam-infected macaques. The specific cellular responses to 47mp were generally less pronounced, with significant differences of peak levels for both Karp-and Gilliam infected macaques as follows; Karp group = BL vs 28 dpi and 6 vs 28 dpi at p = 0.0286 and Gilliam = BL vs 80 dpi and 6 vs 80 dpi at p = 0.0286. The responses against the 47mp of the two groups differed significantly at 28 dpi (p = 0.0238). Interestingly, at 80 dpi the magnitude of IFN-γ-secreting cell responses in the Karp group declined for both peptide pools, while the responses in the Gilliam group gradually increased to the highest levels in this time series.

#### PBMCs isolated from skin at 80 dpi

**Fig 13:**
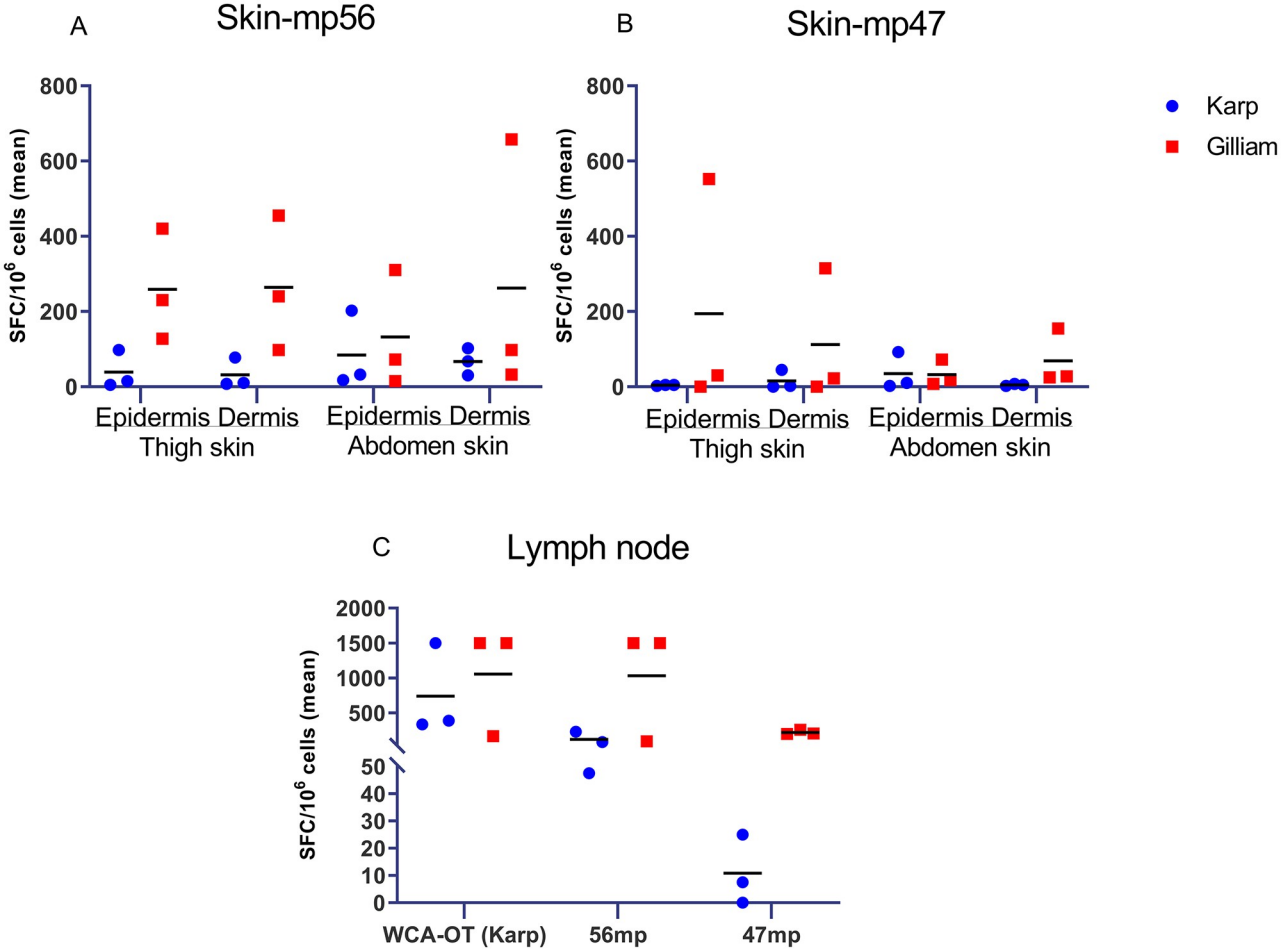
Frequency of antigen-specific IFN-γ producing cells isolated from skin and lymph node following stimulation with *O*. *tsutsugamushi* antigens at 80 days post infection (dpi). Cells were isolated from skin (epidermis of thigh and abdomen, dermis of thigh and abdomen), and inguinal lymph node in n = 3 macaques per group. Antigens used were either a 56-kDa TSA peptide megapool (mp56), a 47-kDa *HtrA* peptide megapool (mp47) or a whole cell antigen *Orientia tsutsugamushi* (WCA-OT Karp) antigen.

The magnitude of IFN-γ-producing T cells were assessed from cells isolated from skin (obtained from thigh and abdomen areas) and inguinal lymph nodes against WCA-OT (Karp), 56-kDa and 47-kDa peptide megapools (Fig 13). The cellular responses from skin (epidermis and dermis) appeared higher in Gilliam-infected macaques for both peptide megapools, but did not reach significant differences when compared to the Karp group due to sample size limitations (Fig 13 and S6 Table). The cells isolated from inguinal lymph nodes in the Gilliam infected macaques also showed higher responses to both 56-kDa and 47-kDa megapools at 80 dpi, but did not reach any significant difference (Fig 5 and S6 Table).

## Discussion

This study further improved the previously evaluated ID challenge rhesus macaque scrub typhus model through incorporation of the Gilliam strain as inoculum. For evaluation and characterization of the Gilliam strain pathogenicity in rhesus macaques, we had to overcome a series of issues; (i) to date no data on Gilliam associated pathogenicity in rhesus macaques is available in literature, although reports from the 1970s suggest this strain to be of high virulence in Silvered-Leaf and cynomolgus macaques [18,23,35,36]; (ii) the limitation of qPCR-based approaches for inoculum characterization, *i*.*e*. quantitation by copy numbers does not reflect functional virulence as inactive pathogens may provide a positive PCR result; (iii) multiple passages of obligate-intracellular *Orientia* spp. in culture may result in virulence reduction, necessitating a “*live*” assessment of an inoculum prior to its application; and importantly (iv) to overcome the standardization difficulties of inocula assessed in mice but applied in macaque models, as we had reached the maximum capacity of functional characterization of Karp strain in outbred mice (10^7.8^ muLD_50_) due to its high murine pathogenicity and lethality [10,33].

### *O*. *tsutsugamushi* Gilliam strain is highly virulent in rhesus macaques

Our data demonstrate that ID infection of rhesus macaques with the *O*. *tsutsugamushi* Gilliam strain results in pronounced pathology highly similar to humans. Gilliam strain inoculation was associated with some distinct advantages in comparison to the Karp strain inoculation: 1) more reliable formation of eschars and more pronounced quantifiable eschar pathology with necrotic centers in rhesus macaques; 2) more distinct manifestation of lymphadenopathy; 3) evident induction of fever especially during bacteremia; 4) useful biochemistry profiles with utility of CRP and liver enzyme measurements; 5) stronger inflammatory markers with higher IFN-γ, IL-1ra, IL-15, IFN-γ/IL-10 and CRP/IL-10 ratios; 6) new evidence of correlations between CRP and IFN-γ with bacterial load; 7) higher mean antibody response titers; and 8) earlier, more pronounced and sustained cell-mediated immune responses. Moreover, these comparative data showed, that a highly lethal Karp dose in mice was substantially inferior to a Gilliam inoculum in inducing pathology in rhesus macaques. These results necessitate repetition to confirm the difference in virulence between the two strains. The findings confirmed our hypothesis that these inocula would lead to similar pathologies in a comparison study, but with the important advantage that with use of the Gilliam strain, a further increase of the inoculum dosage using functional virulence characterization is now possible, in order to develop a lethal NHP model.

### Characterization of two clinically relevant human strains of *O*. *tsutsugamushi*

Limited information is known about the relationship between genotype, antigenicity, clinical course outcome and host defense. As a representative NHP model of the disease, this study illuminates how two predominant strains in the endemic areas correlate with disease severity and host immune responses against scrub typhus. Following the inoculations, strong IFN-γ secreting producing cell responses, humoral immune responses, pro-and anti-inflammatory cytokine productions were observed in both Karp- and Gilliam-infected macaques–these findings provide crucial foundation knowledge for the development of novel therapeutic and vaccine strategies.

To date, no time-course kinetics are available for clinical findings, biomarkers, humoral immune responses, cellular immune responses and immune mediator profiles following the ID inoculation with two highly human pathogenic *O*. *tsutsugamushi* strains; Karp and Gilliam; this study dissected the dynamics of disease progression from initial infection to convalescence and establishment of memory responses [28]. We observed that Gilliam-infected macaques developed earlier systemic infection (characterized by an earlier bacteremia onset with more pronounced lymphadenopathy) compared to the Karp-infected macaques, but that the bacterial clearance (9-days) and median bacterial loads were similar in both groups. These findings suggest that the bacterial dynamics of the two strains are similar in pattern but different in the “time-to-onset” of the bacteremia. This aligns with previous reports in silvered-leaf macaques, where different onsets of bacteremia were described for different strains of *O*. *tsutsugamushi* (Gilliam had an earlier onset of bacteremia when compared to Karp infected animals) and proposed that the duration of the bacteremia tended to be a property of the inoculated strains, but may not correlate with the virulence of the strain [19]. The current scrub typhus challenge model now benefits from an improved inoculum; firstly, the *O*. *tsutsugamushi* Gilliam strain is more pathogenic in rhesus macaques, but less virulent in mice compared to the Karp strain. This adaptation allows for more disease severity with lower bacterial inoculation dosage, and secondly the switch from murine lethal dosage to infectious dosage titrations enables predictable estimations of functional/clinical effects, as well as improved inoculum scalability towards higher doses. By shifting the clinical endpoint spectrum towards higher disease severity, our ID challenge macaque model is now better aligned with the clinical disease course in humans and covers the endpoints required for future vaccine evaluations.

### Biomarker profiles and dynamics

An important new feature in the Gilliam-infection NHP model is the similarity of CRP dynamics to humans; the CRP levels peaking during bacteremia compares to human disease [37–39]. Clinical studies have established that higher CRP levels are associated with host inflammation, innate and adaptive immunity activation and disease severity [40–42]. A retrospective case-control study in Korea, demonstrated that CRP levels associated with poor prognosis in patients with scrub typhus [43]. In this Gilliam-NHP model, tight and robust correlations were observed between CRP and IFN-γ serum levels with bacterial loads over time (Figs 7 and 11).

However, Gilliam-infected macaques did not experience significantly raised liver injury markers throughout the study. Only at 12 dpi did Karp-infected macaques display significantly higher AST plasma levels than the Gilliam-infected macaques. A comparative clinical study of human pathology involving different *O*. *tsutsugamushi* strains in Guangzhou, China, described significantly higher AST levels in humans infected with the Karp strain, compared to Gilliam infections, although the performed strain characterization was limited by the use of partial 56kDa sequences only [44]. The hepatitis in patients with scrub typhus is associated with direct hepatocellular cytopathic damage due to scrub typhus-associated vasculitis and/or perivasculitis [45,46], and appears to relate with severity of disease [47,48]. In this study the elevated AST levels did not reach critical levels for severe infection (>four-fold normal levels) [49] in both Karp- and Gilliam-infected macaques, but was sufficient to serve as a differential factor between the two strains.

### The humoral immune response in *O*. *tsutsugamushi* infection

The role of humoral protection in scrub typhus has not yet been fully clarified with regards to protection against heterologous versus homologous re-exposure [50]. A study in mouse model reported a failure of protection against homologous or heterologous strain of *O*. *tsutsugamushi* following a passive transfer of convalescent sera [51]. In previous NHP studies, the titers of *O*. *tsutsugamushi-*specific antibodies differed among strains, whereby higher titers of total specific antibody from Gilliam-infected macaques were found, but longer persistence was not associated with strain virulence [18,19]. A recent clinical cohort study proposed that a high initial IgG may be associated with a long term higher risk of complicated scrub typhus [52]. In our study, the Gilliam-infected macaques tended to produce higher specific antibody titers in more animals over time when compared to the Karp infected macaques, however this result may be affected also by the inoculum dose. Our study was not designed to dissect the humoral immune response, and IgM and IgG specific antibodies were determined together for general dynamics since differential IgM and IgG antibody responses are well described during the course of disease [52,53]. Since neutralizing antibodies play an important role in vaccine-induced immunity, this model would support multiple-strain long-term studies to characterize the humoral immune response–including sequential re-exposure with different strains and assessment of (cross-)protection using antigenic cartographic analyses *O*. *tsutsugamushi* [54].

### Immune-mediator profiles and dynamics

Upon *O*. *tsutsugamushi* infection, a “cytokine storm” is known to occur in a subgroup of patients with scrub typhus [16,26] and may be associated with severe complications [14,15,27]. A number of studies reported that IL-6, IL-8 [14], IL-10 [15], MCP-1 [14], MIP-1β [14] and TNF [15,17,43] serum levels correlate with disease severity. However, the role of the cytokine network in controlling the infection remains incompletely understood. Previous studies investigated cytokine-mediated immune responses usually at two time points only—the acute- and convalescent phases. In this study, the time-course profiling of immune-mediators was expanded to 6 time points from acute infection to control of the disease (baseline, 6, 12, 18, 28 and 80 dpi) and included two clinically relevant strains allowing detailed dissection and comparisons of immune mediator dynamics and their time association with clinical findings.

In line with human reports, we observed the induction of pro-and anti-inflammatory cytokines relating to the bacteremia time-course curve in infected macaques (Fig 9). Early response cytokines (i.e. first wave at initiation of bacteremia) included IFN-γ, IL-1β, IL-6, IL-18, IL-1ra, IL-10, IL-12 (p40), IL-17a and MCP-1, which all reached peak levels during bacteremia. The major production sources of these cytokines are either dendritic cells (associated with T cell priming: IL-1ra and IL-6, and T cell maturation: IL-12, IL-18) [55], macrophages (M1-type; IL-6, IL-12, and IL-18 and M2 type; IL-10) [56], or endothelial cells (IL-6 and IL-8) [57]. All of these cells are associated with documented intracellular *O*. *tsutsugamushi* infection, reflecting the cellular tropism of *O*. *tsutsugamushi* [30,58]. While the early immune mediators mainly activate innate immune cells, promote IFN-γ production and induce Th1 type responses [59,60], the “second wave” cytokines consisted of IL-8, MIP-1α, MIP-1β, G-CSF, GM-CSF, IL-13, IL-15 and IL-4, TNF and VEGF, all of which peaked after clearance of bacteremia (around 18 dpi). The consequences of counter-balancing effects due to pro- and anti-inflammatory cytokines over time, may associate with either pathogenic or protective roles against *O*. *tsutsugamushi* infection. Further investigations are needed to identify the functional role of these cytokines in scrub typhus pathology.

The cytokine-mediated response profiles were similar in pattern for both Karp-and Gilliam-infected groups, but Karp-infected macaques tended towards higher production of both Th1 and Th2 immune mediators. In contrast, Gilliam-infected macaques developed higher host inflammation with potent Th1-type responses, reflected by early and high IFN-γ serum levels with significantly higher IFN-γ/IL-10 and CRP/IL-10 ratios—suggestive for higher disease severity. These ratios are established disease severity predictors for pulmonary and extrapulmonary tuberculosis [61]. These findings may also suggest different immune mediator pathways stimulated by different constituents / virulence factors of *O*. *tsutsugamushi* bacterial strains and are suggestive that clinical outcomes of scrub typhus are closely associated with the counter balancing effects of the pro- and anti-inflammatory cytokine-mediated responses. Additionally, these findings suggest distinct strain-specific underlying immune mechanisms in response to *O*. *tsutsugamushi* infection [15,60,62,63].

The current study indicates for the first time the potential importance of IL-15 and IL-1ra during *O*. *tsutsugamushi* infection. IL-15 is reported to play a pivotal role in the development, survival and function of NK cells mediated host response [64], and several clinical studies have demonstrated that NK cells from patients with scrub typhus contribute to enhanced intracellular bacterial killing [65,66]. IL-1ra, the receptor antagonist for IL-1, was reported to inhibit the E-selectin, vascular adhesion molecule-1 (VCAM-1) and intercellular adhesion molecule-1 (ICAM-1) expression at the late phase inflammatory responses in *Rickettsia conorii* (a tick-borne rickettsiosis) infection in Human Umbilical Vein Endothelial Cells (HUVEC) line [67] which is one of the key mechanisms of *Rickettsia* spp. to evade host immune responses [68]. Therefore, upregulation of IL-1ra during acute infection may synergize with IL-10 action in limiting host immunopathology, both innate and adaptive host immune responses. TNF levels have been reported as disease severity predictor in scrub typhus patients with very high serum levels during the acute phase of infection [43]. In this study TNF peak concentrations were found when the bacteremia became undetectable (at 18 dpi) in both groups. TNF has an established role in controlling *O*. *tsutsugamushi* growth *in vitro* [69], but TNF production was reported to be inhibited by IL-10-inducing factor in macrophages infected with *O*. *tsutsugamushi* [70]. In this study, we observed peak concentrations of IL-10 during bacteremia suggestive of an anti-inflammatory response dominated by IL-10, which likely links to low TNF production levels during bacteremia. These findings provide supporting information on the critical role of IL-10 in controlling scrub typhus disease severity [14,71]. In conclusion, the cytokine profiles following ID inoculation demonstrate strong pro- and anti-inflammatory Th1 type responses and suggest dysregulation of several host immune defense mechanisms in line with previous studies [14,26,72]. Our study further illuminates how orchestration of the cytokine/chemokine mediated response contributes to disease control and immunopathogenesis in scrub typhus.

### Cell-mediated response dynamics

Similar to our previous study [29], ID infection was followed by a strong IFN-γ-mediated cellular response (measured against whole cell antigen (WCA) for *O*. *tsutsugamushi*), and protection against *O*. *tsutsugamushi* infection is associated with the extent of cell-mediated immune responses [73]. In this study, IFN-γ mediated cellular immune responses correlated strongly with bacterial load (Fig 11), which again associates with disease severity, highlighting these clinically relevant relationships. Further, a long-lasting cellular response was shown as for all three *O*. *tsutsugamushi* derived antigens, we observed preservation of antigen-specific T cell responses in both the Karp- and Gilliam-infected macaques at 80 dpi. Trends toward higher levels of antigen-specific cellular immune responses (measured by 56 kDa and 47 kDa protein megapools) were observed in the Gilliam infected macaques–in blood, skin and lymph node derived immune cells, even after 80 days post-inoculation. These findings suggest a longer or more pronounced duration of cellular memory immune responses induced by the Gilliam strain, arguing in favor of the benefits in applying this strain for future ID challenge studies. Future work will include more in-depth investigation of infected cell phenotypes as the source of IFN-γ secretion, alongside epitope mapping studies to identify which immunogen of the Gilliam strain contributes to longer memory cellular immune responses.

### Inoculum considerations

Inoculum preparation and quantitation approaches all have distinct limitations; PCR does not accurately reflect the proportion of live bacteria, Live/Dead staining does not fully reflect infectiousness of viable bacterial cells, the difficult-to-quantify effect of *in vitro* cell-culture, multiple passages and freeze-thaw cycles on bacterial infectivity and virulence. This is why we opted for a functional biological titration of the inoculum in outbred mice in this study. The utility of the clinical endpoints “infection” and “lethality” in a standardized murine model incorporates all cumulative inherent inaccuracies affecting the final viable infective bacterial proportion in the inoculum. To date, no data on Gilliam strain infection in rhesus macaques is available supporting the estimation of possible differences in the inocula generated, hence the comparative findings in this study are defined (and limited) by the ID50 and LD50 dose titration equivalents determined in outbred mice (S1 Table). The major aim of the study was to characterize a more severe intradermal rhesus macaque model for scrub typhus with potential to reach dosages for a lethal model in the future–this was achieved–and also supports improved identification of correlates of protection in challenge studies. It has to be noted however, that comparative findings relate to the functional inoculum dosages in mice, which demonstrate some variability if other methodologies for quantitation are applied (S1 Table). Clearly, more detailed in-depth comparisons into *Orientia* spp. strain virulence in rhesus macaques would require well-planned studies involving laborious inoculum characterization and quantitation procedures.

## Conclusion

Currently, no data on characterized time-course comparisons of *O*. *tsutsugamushi* strains regarding measures of disease severity and immune response is available. This study compared two highly human pathogenic strains of *O*. *tsutsugamushi* in a validated rhesus macaque model, based on inocula with functional characterization. We provide new evidence in the strain-specificity of host responses in scrub typhus and demonstrated multiple distinct advantages of incorporating the Gilliam strain. ID inoculation with the Gilliam strain associated with higher disease severity in the rhesus macaque model, allowing for improved endpoint measurements and supporting the identification of correlates of protection for future vaccine development.

## Supporting information

S1 Table Inoculation dosages of *O*. *tsutsugamushi* for this study. (DOCX)

S2 Table RISE SCORE (Dermal Draize observation and scoring) observation of the intradermal inoculation lesions for the Karp (n = 4) and Gilliam (n = 4) infected macaques between 0 to 28 dpi. R = Redness (erythema), I = Induration of the skin (infiltration), S = Swelling (edema), E = Eschar formation (necrosis), Ø: Diameters of the recorded lesions are documented in mm.
(DOCX)

S3 Table The time-course kinetics of plasma biochemistry values of Karp (n = 4) and Gilliam (n = 4) strain infected macaques following ID inoculation. All values presented from 0 to 80 dpi.
(DOCX)

S4 Table The anti-*O*. *tsutsugamushi* antibody titers of Karp (n = 4) and Gilliam (n = 4) strain infected macaques following ID inoculation. All values presented from 0 to 80 dpi (ND = not done).
(DOCX)

S5 Table The Median serum concentrations (ρg/ml) of immune mediators determined by Milliplex kit of Karp (n = 4) and Gilliam (n = 4) strain infected macaques following ID inoculation. All values presented from baseline (day -21 before inoculation) to 80 dpi. All data presented are medians (±95% confidence interval CI)
(DOCX)

S6 Table e*x vivo* IFN-γ ELISpot specific to *O*. *tsutsugamushi* antigens (WCA-OT (Karp), 56-kDa megapool and 47-kDa megapool) result from cells isolated from skin at thigh, abdomen areas and inguinal lymph node at 80 dpi of Karp (n = 3) and Gilliam (n = 3) strain infected macaques following ID inoculation. Note: SCF = spot forming cells per million, ND = not done.
(DOCX)
