# Correction: A time-course comparative clinical and immune response evaluation study between the human pathogenic *Orientia tsutsugamushi* strains: Karp and Gilliam in a rhesus macaque (*Macaca mulatta*) model

**DOI:** 10.1371/journal.pntd.0011277

**Published:** 2023-04-12

**Authors:** Manutsanun Inthawong, Piyanate Sunyakumthorn, Sirima Wongwairot, Tippawan Anantatat, Susanna J. Dunachie, Rawiwan Im-Erbsin, James W. Jones, Carl J. Mason, Luis A. Lugo, Stuart D. Blacksell, Nicholas P. J. Day, Piengchan Sonthayanon, Allen L. Richards, Daniel H. Paris

There is an error in the affiliation order for author Manutsanun Inthawong. The correct affiliation order is:

Manutsanun Inthawong^1,2^

**1** Department of Molecular Tropical Medicine and Genetics, Faculty of Tropical Medicine, Mahidol University, Bangkok, Thailand
**2** Department of Veterinary Medicine, United States Army Medical Directorate, Armed Forces Research Institute of Medical Sciences (USAMD-AFRIMS), Bangkok, Thailand
