# Supplementary material for: A time-course comparative clinical and immune response evaluation study between the human pathogenic Orientia tsutsugamushi strains: Karp and Gilliam in a rhesus macaque (Macaca mulatta) model
Source: PLoS Negl Trop Dis. 2022 Aug 4;16(8):e0010611. doi: 10.1371/journal.pntd.0010611 (PMC9352090; doi:10.1371/journal.pntd.0010611)
Supplement: S1 Table — (DOCX) [file pntd.0010611.s001.docx]

**S1 Table**. **Inoculation dosages of *O. tsutsugamushi* for this study**

| **Strains of *O. tsutsugamushi*** | **Dose of *O. tsutsugamushi* per 200 µl in stock solution** | | | |
| --- | --- | --- | --- | --- |
|  | **MuID50 (titer)** | **MuLD50 (titer)** | **Live/Dead Stain (# of live cells)** | **Otsu47 qPCR assay (# of genomic equivalents)** |
| Karp | 1x10^7.8^ | 1x10^7.8^ | 1.35x10^9^ | 3.8x10^8^ |
| Gilliam | 1x10^8.5^ | 1x10^3.6^ | Currently not available | 4.4x10^9^ |

MuID50: Murine infectious dose that infects 50% of mice inoculated

MuLD50: Murine lethal dose that kills 50% of mice inoculated

Titer: Inverse of the highest dilution that infects or kills 50% of mice inoculated
