# Supplementary material for: A time-course comparative clinical and immune response evaluation study between the human pathogenic Orientia tsutsugamushi strains: Karp and Gilliam in a rhesus macaque (Macaca mulatta) model
Source: PLoS Negl Trop Dis. 2022 Aug 4;16(8):e0010611. doi: 10.1371/journal.pntd.0010611 (PMC9352090; doi:10.1371/journal.pntd.0010611)
Supplement: S2 Table — R = Redness (erythema), I = Induration of the skin (infiltration), S = Swelling (edema), E = Eschar formation (necrosis), Ø: Diameters of the recorded lesions are documented in mm. (DOCX) [file pntd.0010611.s002.docx]

**S2 Table**. **RISE SCORE (Dermal Draize observation and scoring) observation of the intradermal inoculation lesions for the Karp (n=4) and Gilliam (n=4) infected macaques between 0 to 28 dpi**.

R = Redness (erythema), I = Induration of the skin (infiltration), S = Swelling (edema), E = Eschar formation (necrosis), Ø: Diameters of the recorded lesions are documented in mm.

**Dermal Reaction Scoring Code:**

0 = Absence/ no evidence of skin redness, swelling or induration or any lesion

*Redness/erythema (Ø)*

R1 = Mild skin redness (pink skin in color)

R2 = Moderate skin redness (red skin in color)

R3 = Severe skin redness (dark red in color)

*Induration/infiltration*

I1 = Mild induration (hardening) is palpated as a hard area with a diameter < 5 mm

I2 = Moderate induration, palpated as a larger, hardened area with a diameter 5 – 10 mm

I3 = Severe induration, the whole area is hardened and obviously enlarged with a diameter >10 mm

*Swelling/edema*

S1 = skin slightly swollen, affected area is Ø < 5 mm

S2 = skin markedly swollen, the affected area is Ø 5 – 10 mm

S3 = skin obviously swollen, the affected area is Ø > 10 mm

*Eschar/necrosis*

E1 = Indurated papule, color livid-deep red

E2 = Excoriated papule with small black scab/crust

E3 = Demarked excoriation with crust, raised indurated border
