# Supplementary material for: A time-course comparative clinical and immune response evaluation study between the human pathogenic Orientia tsutsugamushi strains: Karp and Gilliam in a rhesus macaque (Macaca mulatta) model
Source: PLoS Negl Trop Dis. 2022 Aug 4;16(8):e0010611. doi: 10.1371/journal.pntd.0010611 (PMC9352090; doi:10.1371/journal.pntd.0010611)
Supplement: S3 Table — All values presented from 0 to 80 dpi. (DOCX) [file pntd.0010611.s003.docx]

**S3 Table.** **The time-course kinetics of plasma biochemistry values of Karp (n=4) and Gilliam (n=4) strain infected macaques following ID inoculation**; values represent post-inoculation days – ranging from Day 0 to Day 80.
