# Supplementary material for: A time-course comparative clinical and immune response evaluation study between the human pathogenic Orientia tsutsugamushi strains: Karp and Gilliam in a rhesus macaque (Macaca mulatta) model
Source: PLoS Negl Trop Dis. 2022 Aug 4;16(8):e0010611. doi: 10.1371/journal.pntd.0010611 (PMC9352090; doi:10.1371/journal.pntd.0010611)
Supplement: S4 Table — All values presented from 0 to 80 dpi (ND = not done). (DOCX) [file pntd.0010611.s004.docx]

**S4 Table**. **The anti-*O. tsutsugamushi* antibody titers of Karp (n=4) and Gilliam (n=4) strain infected macaques following ID inoculation;** all values presented from 0 to 80 dpi (ND=not done, dpi=days post inoculation, ID=intradermal).

| **Day post infection (dpi)** | **Antibody titer** | | | | | | | |
| --- | --- | --- | --- | --- | --- | --- | --- | --- |
|  | **Karp** | | | | **Gilliam** | | | |
|  | **#1** | **#2** | **#3** | **#4** | **#1** | **#2** | **#3** | **#4** |
| **0** | 0 | 0 | 0 | 0 | 0 | 0 | 0 | 0 |
| **6** | 100 | 100 | 0 | 200 | 100 | 0 | 0 | 0 |
| **12** | 3200 | 3200 | 3200 | 1600 | 12800 | 3200 | 6400 | 1600 |
| **18** | 12800 | 12800 | 25600 | 3200 | 25600 | 25600 | 6400 | 25600 |
| **28** | 12800 | 12800 | 3200 | 3200 | 25600 | 25600 | 6400 | 25600 |
| **80** | 6400 | 3200 | 3200 | ND | 6400 | 6400 | 6400 | ND |
