# Supplementary material for: A time-course comparative clinical and immune response evaluation study between the human pathogenic Orientia tsutsugamushi strains: Karp and Gilliam in a rhesus macaque (Macaca mulatta) model
Source: PLoS Negl Trop Dis. 2022 Aug 4;16(8):e0010611. doi: 10.1371/journal.pntd.0010611 (PMC9352090; doi:10.1371/journal.pntd.0010611)
Supplement: S5 Table — All values presented from baseline (day -21 before inoculation) to 80 dpi. All data presented are medians (±95% confidence interval CI) (DOCX) [file pntd.0010611.s005.docx]

**S5 Table. The median serum concentrations (ρg/ml) of immune mediators determined by Milliplex kit of Karp (n=4) and Gilliam (n=4) strain infected macaques following ID inoculation**; all values presented from baseline (Day -21 before inoculation) to 80 dpi. All data presented are medians (±95% confidence interval CI)


Note: “0” signifies concentration below the assay detection limit; dpi=days post inoculation; ID=intradermal.
