# Supplementary material for: A time-course comparative clinical and immune response evaluation study between the human pathogenic Orientia tsutsugamushi strains: Karp and Gilliam in a rhesus macaque (Macaca mulatta) model
Source: PLoS Negl Trop Dis. 2022 Aug 4;16(8):e0010611. doi: 10.1371/journal.pntd.0010611 (PMC9352090; doi:10.1371/journal.pntd.0010611)
Supplement: S6 Table — Note: SCF = spot forming cells per million, ND = not done. (DOCX) [file pntd.0010611.s006.docx]

**S6 Table.** **Overview of *ex vivo* IFN-γ ELISpot results specific to *O. tsutsugamushi* antigens (WCA-OT Karp, 56-kDa megapool and 47-kDa megapool) using cells isolated from skin (thigh and abdomen) and inguinal lymph nodes at 80 dpi of Karp (n=3) and Gilliam (n=3) strain infected macaques following ID** **inoculation.**

Note: SCF = spot forming cells per million; ND = not done; dpi=days post inoculation; ID=intradermal.
